# Characterizing neuroinflammation and identifying prenatal diagnostic markers for neural tube defects through integrated multi-omics analysis

**DOI:** 10.1186/s12967-024-05051-8

**Published:** 2024-03-09

**Authors:** Wenshuang Wang, Yanhong Ji, Zhexu Dong, Zheran Liu, Shuang Chen, Lei Dai, Xiaolan Su, Qingyuan Jiang, Hongxin Deng

**Affiliations:** 1 Department of Biotherapy, Cancer Center and State Key Laboratory of Biotherapy, West China Hospital, Sichuan University, Chengdu, China; 2 Department of Obstetrics, Sichuan Provincial Hospital for Women and Children, Chengdu, China

**Keywords:** Neural tube defects (NTDs), Multi-omics analysis, Neuroinflammation, JAK-STAT signaling pathway, Macrophage polarization, Prenatal diagnosis

## Abstract

**Background:**

Neural Tube Defects (NTDs) are congenital malformations of the central nervous system resulting from the incomplete closure of the neural tube during early embryonic development. Neuroinflammation refers to the inflammatory response in the nervous system, typically resulting from damage to neural tissue. Immune-related processes have been identified in NTDs, however, the detailed relationship and underlying mechanisms between neuroinflammation and NTDs remain largely unclear. In this study, we utilized integrated multi-omics analysis to explore the role of neuroinflammation in NTDs and identify potential prenatal diagnostic markers using a murine model.

**Methods:**

Nine public datasets from Gene Expression Omnibus (GEO) and ArrayExpress were mined using integrated multi-omics analysis to characterize the molecular landscape associated with neuroinflammation in NTDs. Special attention was given to the involvement of macrophages in neuroinflammation within amniotic fluid, as well as the dynamics of macrophage polarization and their interactions with neural cells at single-cell resolution. We also used qPCR assay to validate the key TFs and candidate prenatal diagnostic genes identified through the integrated analysis in a retinoic acid-induced NTDs mouse model.

**Results:**

Our analysis indicated that neuroinflammation is a critical pathological feature of NTDs, regulated both transcriptionally and epigenetically within central nervous system tissues. Key alterations in gene expression and pathways highlighted the crucial role of STATs molecules in the JAK-STAT signaling pathway in regulating NTDs-associated neuroinflammation. Furthermore, single-cell resolution analysis revealed significant polarization of macrophages and their interaction with neural cells in amniotic fluid, underscoring their central role in mediating neuroinflammation associated with NTDs. Finally, we identified a set of six potential prenatal diagnostic genes, including *FABP7*, *CRMP1*, *SCG3*, *SLC16A10*, *RNASE6* and *RNASE1*, which were subsequently validated in a murine NTDs model, indicating their promise as prospective markers for prenatal diagnosis of NTDs.

**Conclusions:**

Our study emphasizes the pivotal role of neuroinflammation in the progression of NTDs and underlines the potential of specific inflammatory and neural markers as novel prenatal diagnostic tools. These findings provide important clues for further understanding the underlying mechanisms between neuroinflammation and NTDs, and offer valuable insights for the future development of prenatal diagnostics.

**Supplementary Information:**

The online version contains supplementary material available at 10.1186/s12967-024-05051-8.

## Background

Neural development is a complex and highly orchestrated process that gives rise to the intricate architecture of the nervous system [1]. The formation of the neural tube, the embryonic precursor of the brain and spinal cord, represents a critical event during early embryogenesis [2]. Any disruptions in this process can lead to severe congenital malformations known as neural tube defects (NTDs), which encompass conditions such as spina bifida, encephalocele, and anencephaly [3]. NTDs are a significant public health concern, contributing to substantial morbidity and mortality worldwide. The prevalence of NTDs varies by geographical region and population. On a global scale, the estimated average prevalence of NTDs is approximately 1 in 1,000 pregnancies [4]. The etiology of NTDs is multifactorial, involving both genetic and environmental factors, and their precise pathogenesis remains incompletely understood [5].

Neuroinflammation refers to the inflammatory response in the nervous system, typically resulting from damage to neural tissues due to various reasons, including infections, trauma, autoimmune diseases, or other conditions [6]. During neuroinflammation, immune cells such as microglia and lymphocytes release inflammatory mediators that can have detrimental effects on neurons and other neural cells [7]. Prolonged neuroinflammation is believed to be associated with many neurological disorders, including Parkinson's disease [8], Alzheimer's disease [9], and multiple sclerosis [10]. A recent study has identified a series of immune-related genes in human NTDs [11]. Similarly, upregulated inflammation-related processes were found in spinal cords from rats with NTDs [12]. Additionally, the use of scRNA-Seq has revealed the origin and heterogeneity of cellular contents in the cultured amniotic fluid (AF) from human fetuses with NTDs and briefly mentioned the existence of immune cells [13]. However, the immune and neural cell landscapes, gene regulatory networks (GRNs), and underlying mechanisms linking neuroinflammation to the pathogenesis, development, and progression of NTDs have not been extensively investigated.

Mouse models provide a valuable tool for investigating the molecular mechanisms underlying NTDs due to their genetic similarity to humans and the ability to control for environmental factors. By inducing specific genetic mutations [14] or manipulating certain environmental factors, such as folate deficiency [15], sodium valproate induction [16], and retinoic acid induction [17], mouse models can be created to mimic different types of NTDs observed in humans. The use of these mouse NTDs models has allowed for the identification of key genes, biomarkers, and signaling pathways involved in NTDs development [18–20].

Prenatal diagnosis of NTDs plays a crucial role in the management and counseling of affected pregnancies [21]. Current diagnostic modalities, such as maternal serum alpha-fetoprotein screening, fetal ultrasonography, and amniocentesis, have limitations in terms of sensitivity, specificity, and invasiveness [21–23]. A recent study has identified immune-related genes as diagnostic biomarkers of NTDs in fetal tissues [11]. However, these biomarkers lack practical application because the detection of fetal tissue during pregnancy is challenging and carries risks. Therefore, there is a pressing need for the development of novel, non-invasive prenatal diagnostic approaches that can accurately detect NTDs and provide valuable prognostic information to expectant parents.

In this study, we integrated nine public datasets from transcriptomics, epigenomics, and single-cell transcriptomics of human and mouse to comprehensively dissect the complex molecular and cellular networks governing the interplay between neuroinflammation and NTDs. We identified neuroinflammation as the significant pathological feature in both central nervous system (CNS) and AF form fetuses with NTDs. Moreover, we revealed that macrophages, exhibiting distinct polarization, were responsible for the NTDs-associated neuroinflammation in AF. Finally, with a focus on prenatal diagnostic markers, we proposed a conserved “M + N” (Macrophage + Neural) method that utilizes AF samples to provide an indication of the severity of NTDs after validation in a RA-induced mouse NTDs model.

## Methods and materials

### RA (retinoic acid)-induced NTDs mouse animal model

SPF grade C57BL/6J male and female mice (GemPharmatech, Nanjing, China), aged between 10 and 16 weeks, were utilized to establish the NTDs mouse model. At E7.5, pregnant dams were gavaged with 16 mg/kg all trans-retinoic acid (RA) (Sigma Aldrich, St. Louis, MO, USA) solubilized in corn oil or an equivalent volume of corn oil only. At E13.5, pregnant dams were euthanized, and fresh AF samples were collected from the fetuses using a syringe. The collected samples were then immediately snap-frozen in liquid nitrogen for subsequent analysis.

### Real-time quantitative PCR (qPCR)

Total mRNA was extracted from mouse AF using the Trizol Reagent, following the manufacturer's isolation protocol (Thermo Fisher, MA). Subsequently, 1 μg of RNA was used to synthesize cDNA using the PrimeScript™ RT reagent Kit (Takara, Japan), following the manufacturer’s instructions. The expression of specific genes was determined using SYBR Green master mix (Takara, Japan). *Gapdh* was utilized as the housekeeping gene, and the 2^−△△Ct^ method was employed for data analysis. The sequences of all primers used for qPCR are provided in Additional file 10: Table S1.

### Differentially expressed genes (DEGs) analysis

R (version 4.2.1) was used for all data analysis. The R package limma (version 3.52.4) [24] was employed for microarray data analysis, while the R package DESeq2 (version 1.36.0) [25] was utilized for high-throughput RNA-seq data analysis. In the case of microarray data, the avereps function was used to correct within-array replicate probes, and missing expression data was imputed using the impute.knn function. Subsequently, the microarray data was normalized between samples using the normalizeBetweenArrays function. DEGs were then identified using the contrasts.fit and eBayes functions. For high-throughput RNA-seq data, the differential matrix was constructed using the DESeqDataSetFromMatrix function, and DEGs were identified with the DESeq function. In the analysis of brain and spinal cord tissues, a threshold of P value < 0.05 and |log2FC|> 0.5 was applied to identify DEGs. For AF, an absolute value of log2FC greater than 1 was used as the threshold to identify DEGs. The pseudobulk-seq analysis was performed using a method described previously [26].

### Protein–protein interaction (PPI) analysis

The protein–protein interaction (PPI) analysis was conducted using the STRING database [27] (https://string-db.org/) with DEGs as input. Subsequently, the results underwent hub gene analysis in the CytoHubba app and were further analyzed and visualized in Cytoscape (version 3.9.1) [28].

### Gene function enrichment analysis

Gene function enrichment analysis was carried out using the R package clusterProfiler (version 4.7.1.001) [29]. Prior to the analysis, the symbols of DEGs were converted into ENTREZID IDs using the bitr function. Enrichment analysis encompassed KEGG (Kyoto Encyclopedia of Genes and Genomes), GO (Gene Ontology), and GSEA (Gene Set Enrichment Analysis) and was conducted using the enrichKEGG, enrichGO, gseKEGG, and gseGO functions, respectively. The results of the enrichment analysis were visualized using the emapplot function.

### Differentially DNA methylated regions (DMRs) analysis

Differentially DNA methylated regions (DMRs) were analyzed using the R package ChAMP (version 2.26.0) [30], specifically employing the 450k analysis methods. The input data, in the form of a matrix of β values, underwent filtering using the champ.filter function and subsequent normalization using the champ.norm function. DMRs were identified using the champ.DMP function. DMRs showing statistical significance with a P-value < 0.05 and an absolute difference in β values (|Δβ|) greater than 0.10 were selected for further functional enrichment analysis.

### Integrated transcriptome and methylome to construct GRNs

The integration of transcriptome and methylome data was conducted using the R package ELMER (version 2.20.0) [31]. A MAE object was constructed by normalizing the transcriptome matrix and a β value methylation matrix. A comparison of methylation levels for all distal probes between the NTDs group and the normal group was carried out, selecting methylation sites with a P-value < 0.05 and |Δβ|> 0.1. Subsequently, the correlation between the methylation level of the distal probe and the expression level of the target gene was analyzed to establish probe-gene pairs. Enrichment analysis was performed using the probes included in the probe-gene pairs to identify enriched motifs. The TF binding motif database was then employed to predict the TFs binding to these motifs. Finally, the relationship between the identified motifs and the upstream TFs was screened to determine the TFs with regulatory effects.

### GRNs analysis in bulk- and scRNA-seq

The NetAct (version 1.0.6) [32] R package was utilized for analyzing GRNs in bulk-seq data. The normalized matrix data was used as input, and DEGs were identified using the RNAseqDegs_limma function. Following this, TFs were selected through the TF_Selection function, and their activities were calculated using the TF_Activity function. For the analysis of GRNs in scRNA-seq data, the SCENIC (version 1.3.1) [33] R package was employed. The analysis commenced with preprocessing the scRNA-seq data, which involved filtering out low-quality cells, normalizing gene expression values, and identifying highly variable genes. Subsequently, a co-expression analysis was conducted to identify clusters or modules of genes with similar expression patterns across cells. Motif enrichment analysis was then performed to identify enriched TF binding motifs within the gene promoters of each gene cluster or module. Finally, utilizing the GENIE3 algorithm, the GRNs were inferred by calculating the direct regulatory effect of TFs on target genes, leveraging their expression levels.

### Cell trajectory analysis for scRNA-seq

The cell trajectory analysis using the R package monocle (version 2.14.0) [34] involved using the matrix and metadata of processed scRNA-seq data as input to construct a CellDataSet object. The top 20 markers of each subcluster were used to order the cells along the trajectory. Additionally, a DDRTree method was employed for dimension reduction analysis. Visualization of the trajectory and gene expression dynamics was achieved through plotting functions, providing insights into the underlying biological processes.

### Cell communication analysis

The CellChat (version 1.6.1) [35] R package was utilized for cell communication analysis. Initially, the scRNA-seq data underwent standard quality control measures and normalization methods. Subsequently, the identifyOverExpressedInteractions function was employed to identify expressed ligand-receptor pairs in each cell type. Following this, the computeCommunProb function in CellChat was used to calculate the communication scores between each pair of cell types. Further analysis included the calculation of communication results for all ligand-receptor interactions associated with each signaling pathway using the computeCommunProbPathway function. To visualize the strength and directionality of the interactions between different cell types, a network diagram was generated utilizing the netVisual_aggregate function. Additionally, the netVisual_bubble function was used to visualize the ligand-receptor pairs.

### Integrating bulk- and scRNA-seq data

The integration of bulk- and scRNA-seq data was carried out using the R package Scissor (version 2.0.0) [36]. For this integration, the input consisted of processed scRNA-seq data with dimensionality reduction information and normalized bulk-seq data. The selection of the alpha value was based on ensuring that the percentage of selected cells was closest to 30%.

### scRNA-seq data analysis

The analysis of scRNA-seq data was performed using the R package Seurat (version 4.3.0) [37], involving several key steps. Initially, the raw matrix was read using the Read10X function, followed by quality control measures to ensure that the percentage of mitochondrial genes per cell was below 20% and selecting genes with a feature count greater than 1500. Cell cycle scoring was performed using the CellCycleScoring function, and doublets were identified using the R package DoubletFinder (version 2.0.3) [38]. To address the potential presence of ambient RNA contamination, we employed the R package decontX (version 0.99.3) [39], and removed any cells with a contamination value exceeding 0.2. Subsequently, normalization, identification of highly variable genes, and data scaling were performed using the SCTransform function. Regression analysis was conducted to account for mitochondrial gene expression and cell cycle effects. Principal Component Analysis (PCA) reduction was performed, retaining 50 dimensions. Sample integration was carried out using the R package harmony (version 0.1.0) [40]. UMAP (Uniform Manifold Approximation and Projection) dimensionality reduction was then applied using 50 dimensions, followed by cell clustering using the FindClusters function with a resolution of 1.2. All markers used for cluster definition were generated using the FindAllMarkers function. Finally, gene expression density was visualized using the Nebulosa R package (version 1.6.0) [41].

### Statistical analysis

In the study, the data were expressed as mean ± standard error of the mean (SEM). Statistical significance was determined using the Student’s t-test and analysis of variance (ANOVA). A P-value less than 0.05 was considered significant, denoted as *P < 0.05; **P < 0.01; ***P < 0.001, while non-significant results were indicated as N.S. It's noted that GraphPad Prism 9.0 software was utilized to conduct all statistical analyses in the study, ensuring robust and widely recognized statistical methodologies were applied.

## Results

### Differentially expressed genes (DEGs) analysis in different tissues of NTDs

**Fig. 1:**
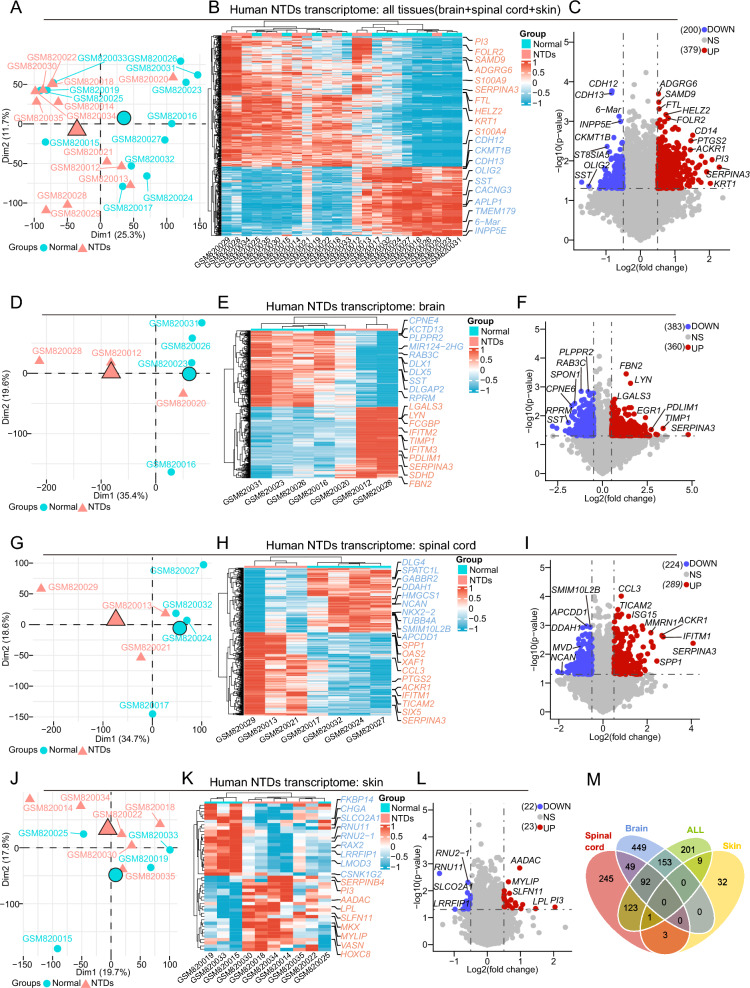
DEGs analysis in various tissues of human NTDs transcriptome. **A**, **D**, **G**, **J** PCA plot depicting all samples, as well as brain, spinal cord, and skin tissue samples involved in DEGs analysis, respectively. **B**, **E**, **H**, **K** Heatmaps illustrating DEGs in all, brain, spinal cord, and skin tissue samples, respectively, with the top DEGs highlighted. **C**, **F**, **I**, **L** Volcano plots visualizing DEGs in all, brain, spinal cord, and skin tissue samples, respectively, with the top DEGs highlighted. **M** Venn diagram displaying common DEGs across different tissues

All data analysis and experimental validation for this study are illustrated in the schematic diagram (Additional file 1: Fig. S1). First, we analyzed the human transcriptomic data of 12 NTDs and 12 normal controls in the second trimester obtained from GEO with accession number GSE33111 (https://www.ncbi.nlm.nih.gov/geo/query/acc.cgi?acc=GSE33111). Given the high cellular heterogeneity during development, we compared the different tissues of NTDs with those of normal controls to accurately identify differentially expressed genes (DEGs). The top 10 DEGs, ranked by log2FC and p-value, were visualized in heatmaps and volcano plots for each group (Fig. 1A–L). Notably, the brain group exhibited the highest number of DEGs, with 383 down-regulated genes and 360 up-regulated genes (Fig. 1F). In contrast, the skin group showed the fewest DEGs, with only 22 down-regulated genes and 23 up-regulated genes (Fig. 1L). Additionally, the all tissues group shared 245 and 216 DEGs with the brain group and the spinal cord group, respectively (Fig. 1M). Furthermore, the brain group and the spinal cord group shared 141 DEGs (Fig. 1M). However, the skin group had only ten and four DEGs in common with the all tissues group and the spinal cord group, respectively (Fig. 1M). These findings suggest that the DEGs associated with NTDs are predominantly located in the CNS, including brain and spinal cord, thus justifying a focused subsequent analysis on these two tissues.

### Identifying neuroinflammation as a notable pathological feature in NTDs

**Fig. 2:**
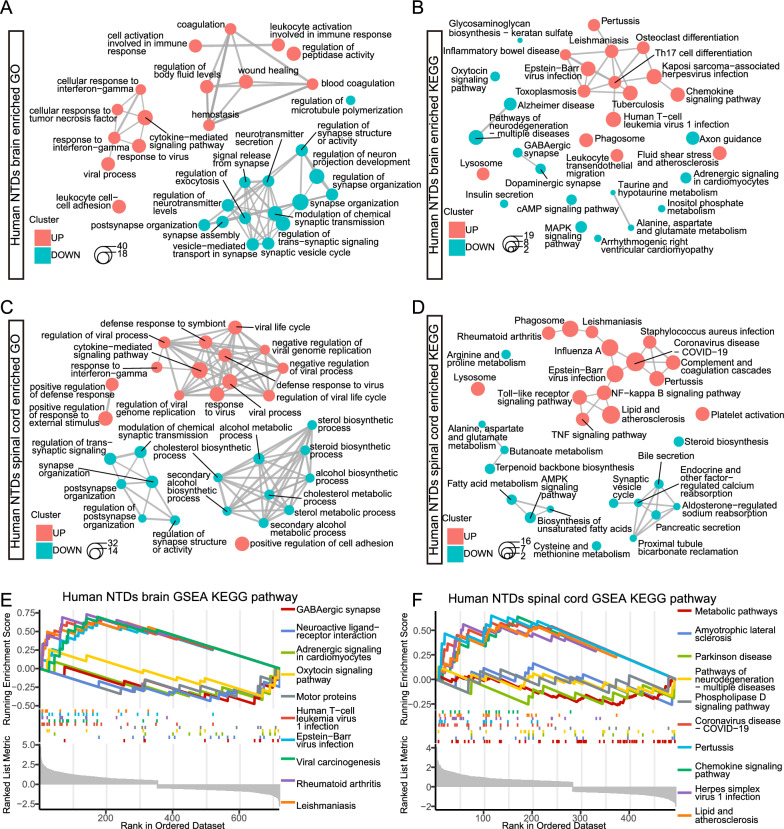
Gene function enrichment analysis of DEGs in human NTDs transcriptome. **A**, **B** Visualization of GO and KEGG enrichment of DEGs in the brain using emapplot, respectively. **C**, **D** Visualization of GO and KEGG enrichment of DEGs in the spinal cord using emapplot, respectively. **E**, **F** Visualization of GSEA for the enriched KEGG pathways in the brain and spinal cord, respectively

To elucidate the function of the DEGs identified in the brain and spinal cord, we conducted GO and KEGG enrichment analyses, along with GSEA pathway analysis. The up-regulated genes were notably enriched in immune-related processes such as leukocyte activation, cytokine-mediated signaling, and response to interferon-gamma (Fig. 2A, C). Furthermore, immune-related pathways such as the chemokine signaling pathway, NF-kappa B signaling pathway, and TNF signaling pathway exhibited significant enrichment among the up-regulated genes (Fig. 2B, D). In contrast, the down-regulated genes were associated with processes related to neurotransmitters, synapses, and the MAPK signaling pathway (Fig. 2A–D). Interesting, we also observed remarkable enrichment in metabolic processes such as cholesterol, sterol, taurine, fatty acids metabolism, and the AMPK signaling pathway among the down-regulated genes (Fig. 2C, D). Similar enrichment patterns were identified in the GSEA pathway analysis (Fig. 2E, F). These results indicate that neuroinflammation is a notable pathological feature of NTDs, while diverse metabolic processes are implicated in the development of the brain and spinal cord.

### NF-kappa B and JAK-STAT signaling uncovered as the key pathways involved in NTDs-associated neuroinflammation

**Fig. 3:**
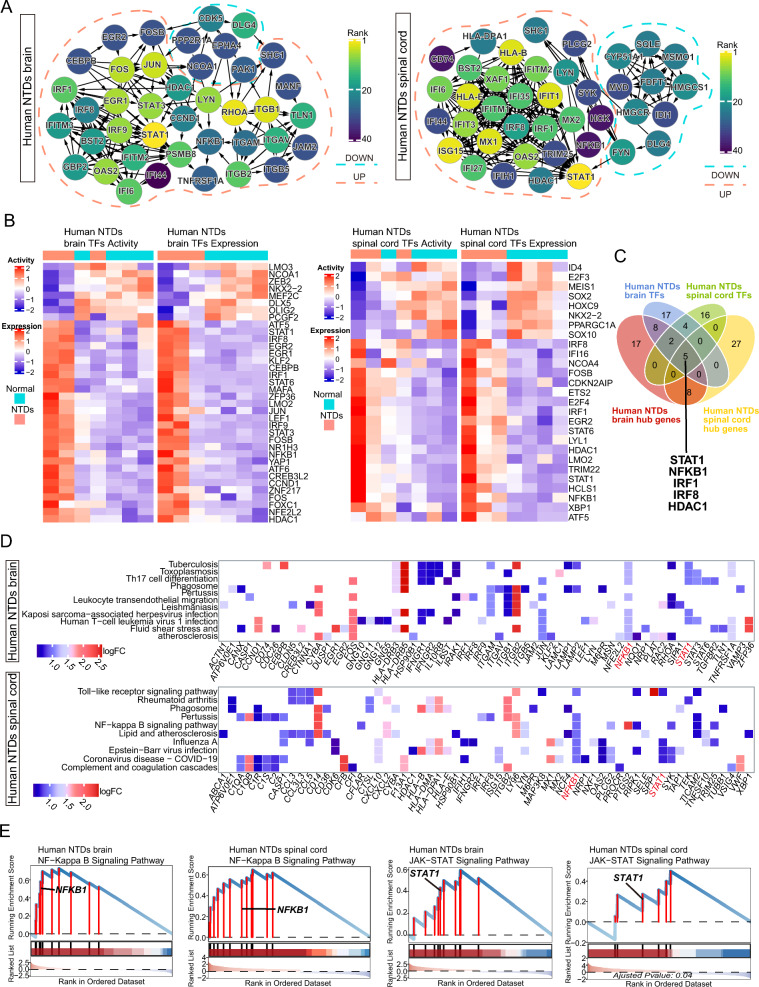
Hub genes and TFs in the brain and spinal cord of human NTDs transcriptome. **A** PPI network of hub genes in the brain and spinal cord of human NTDs transcriptome. **B** NetAct analysis of TF expression and activity in the brain and spinal cord of human NTDs transcriptome. **C** Venn diagram illustrating the intersection of TFs and hub genes in both the brain and spinal cord of human NTDs transcriptome. **D** Heatmap displaying the top 10 pathways in the brain and spinal cord of human NTDs transcriptome, highlighting the involved DEGs. **E** GSEA plot depicting the NF-Kappa B and JAK-STAT signaling pathways in the brain and spinal cord of human NTDs transcriptome

To identify the hub genes involved in NTDs, we constructed the protein–protein interaction (PPI) network for the DEGs of the brain and spinal cord, respectively. The top 40 hub genes identified by cytoHubba displayed a preference for NTDs in both brain and spinal cord (Fig. 3 A). Not surprisingly, the up-regulated hub genes were immune-related, such as *STAT1*, *IRF8* and *NFKB1*, in both brain and spinal cord. Notably, the interaction network for down-regulated hub genes involved in cholesterol metabolism (*HMGCS1*, *FDFT1* and *SQLE*) was significantly enhanced in the spinal cord (Fig. 3A). Furthermore, we observed differential activity in 36 transcriptional factors (TFs) in the brain and 27 TFs in the spinal cord (Fig. 3B). Additionally, these two tissues shared a common set of five genes (*STAT1*, *NFKB1*, *IRF1*, *IRF8*, and *HDAC1*) among the identified TFs and hub genes (Fig. 3C). Among these genes, *NFKB1* and *STAT1* were implicated in most of the top 10 pathways mentioned above (Fig. 3D). NFKB1 and STAT1 are key regulatory proteins involved in the NF-kappa B and JAK-STAT signaling pathways, respectively, which are associated with immune and inflammatory responses [42, 43]. Interestingly, the cross-talk between components of the JAK-STAT signaling pathway and the NF- kappa B signaling pathway is extensive [44]. Further GSEA analysis confirmed the significant upregulation of the NF-kappa B and JAK-STAT signaling pathway associated with NTDs in the brain and spinal cord (Fig. 3 E). These results indicate that the NF-kappa B and JAK-STAT pathways play important roles in neuroinflammation related to NTDs.

### Unveiling transcriptionally and epigenetically regulated neuroinflammation in NTDs

**Fig. 4:**
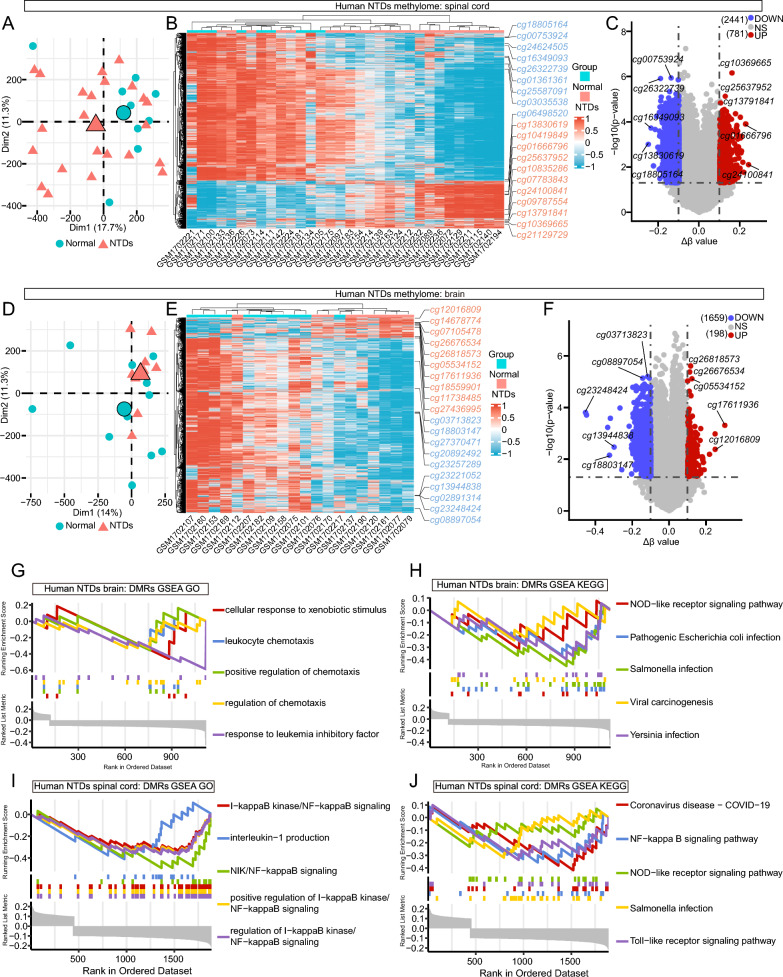
Analysis of DMRs in different tissues of human NTDs methylome. **A**, **D** PCA plots illustrating spinal cord and brain tissue samples involved in DMRs analysis, respectively. **B**, **E** Heatmaps displaying the DMRs in the spinal cord and brain of NTDs methylome, respectively, with the top DMRs being shown. **C**,** F** Volcano plots visualizing DMRs in the spinal cord and brain, respectively, with the top DMRs being shown. **G**, **I** GSEA GO analysis of genes corresponding to DMRs in the spinal cord and brain of NTDs methylome, respectively. **H**, **J** GSEA KEGG analysis of genes corresponding to DMRs in the spinal cord and brain of NTDs methylome, respectively

Folic acid, serving as a methyl donor, is involved in DNA methylation [45]. Sufficient intake of folic acid helps maintain normal DNA methylation levels, which may contribute to reducing the risk of NTDs [46]. Therefore, we investigated the methylome data, categorized by different tissues, from human NTDs during the second trimester (GEO, GSE69502) [47]. In spinal cord tissues (23 NTDs vs. 9 normal controls), 781 up-regulated and 2441 down-regulated differentially DNA methylated regions (DMRs) were identified (Fig. 4A–C). Similarly, in brain tissues (9 NTDs vs. 11 normal controls), 198 up-regulated and 1659 down-regulated DMRs were observed (Fig. 4D–F). The prevalence of down-regulated DMRs surpassed that of up-regulated ones, indicating the significant role of hypomethylation in NTDs progress. The GSEA analysis of genes corresponding to the DMRs revealed enrichment of immune-related processes and pathways in the down-regulated DMRs. (Fig. 4G–J). Specifically, several NF-kappa B signaling-related GO and KEGG terms were identified in the spinal cord (Fig. 4I, J).

**Fig. 5:**
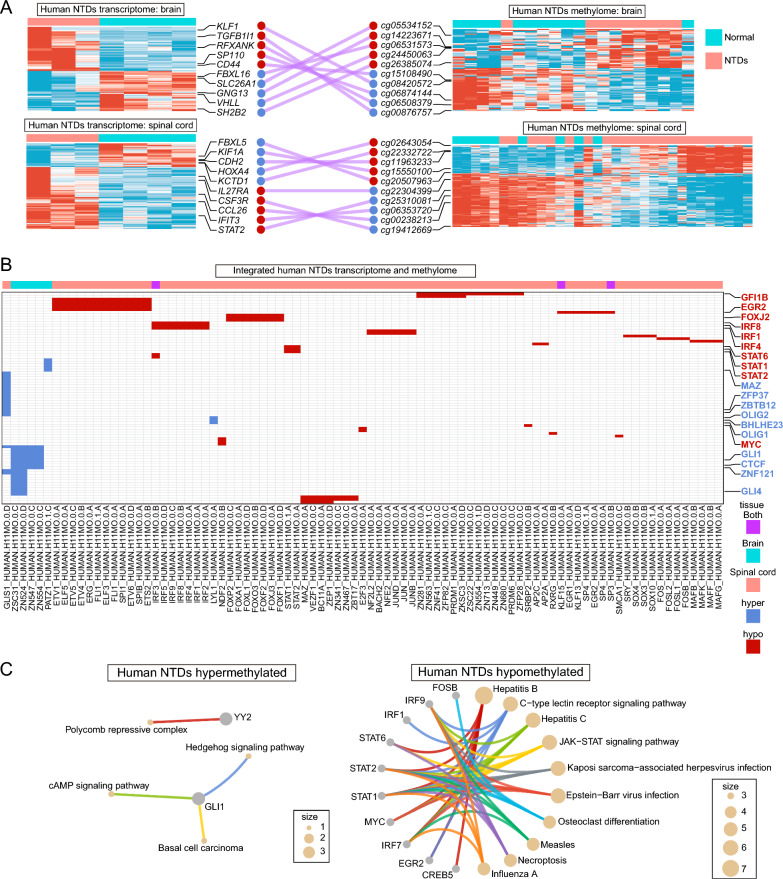
GRNs constructed by integrating human NTDs transcriptome and methylome. **A** Heatmap visualizing the gene-methylated region pairs in the brain and spinal cord, with representative pairs displayed. **B** Heatmap displaying the enriched motifs and corresponding TFs in the spinal cord and brain of NTDs. **C**, **D** GSEA KEGG analysis of hypomethylated and hypermethylated TFs, respectively

To further elucidate the GRNs, we employed ELMER to integrate the transcriptome and methylome data described above. Specifically, we focused on the analysis of methylated regions-gene expression pairs in promoter and enhancer regions. We observed a negatively correlated expression pattern in these pairs, revealing distinct neural (*FBXL16*, *SH2B2*, *KIF1A*, and *HOXA4*) or immune (*IFIT3*, *STAT2*, *IL27RA*, *SP110*, and *CSF3R*) signatures in both brain and spinal cord tissues (Fig. 5A). By utilizing these pairs, we identified motifs for TFs binding and determined the most enriched TFs binding to these motifs. The hypomethylation-associated motifs enriched TFs, such as STAT2, STAT1, STAT6, IRF4, IRF1, IRF8, EGR2, and GFI1B, were markedly linked to immune processes (Fig. 5B, C). Among these TFs, a series of STATs molecules (STAT1, STAT2, STAT6) in the JAK-STAT signaling pathway were observed (Fig. 5B). On the other hand, the hypermethylation-associated motifs enriched TFs, including OLIG1, BHLHE23, OLIG2, ZBTB12, and ZFP37, were evidently associated with nervous system development (Fig. 5B, C). These results suggest that the neuroinflammation of the nervous system is regulated at both the transcriptional and epigenetic levels in NTDs.

### Characterizing the single-cell resolution landscapes of AF-derived neural and immune cells from fetuses with NTDs

**Fig. 6:**
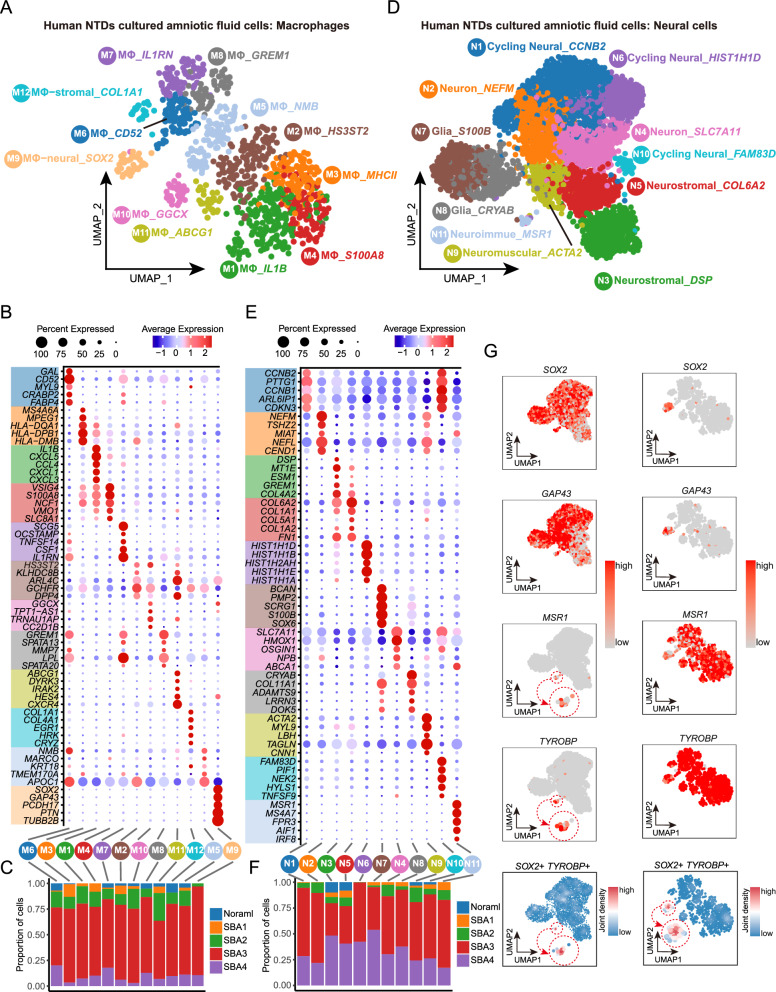
The single-cell resolution profiling of AF-derived neural and immune cells from human fetuses with NTDs **A**, **D** UMAP-based dimension reduction analysis of reclustering macrophage and neural cells, respectively. M and MΦ: Macrophage, N: neural cells. **B**, **E** Display of the top five markers of each subtype in macrophage and neural cells using dot plots, respectively. **C**, **F** Proportion of cells from different samples in each subtype of macrophage and neural cells, respectively. SBA: Spina Bifida. **G** Scatter and density plots of macrophage and neural cell marker genes

To obtain more precise details about NTDs, we undertook a reanalysis of the scRNA-seq data derived from cultured human AF obtained from fetuses with spina bifida (GEO, GSE206696, 4 NTDs vs. 1 normal control) [13] (Additional file 2: Fig. S2 A, D). We observed a significant increase in neural (neural and glial) and immune cells in NTDs compared to normal samples, as previously described (Additional file 2: Fig. S2 B, C). To further elucidate the cellular heterogeneity within these two clusters, which was not clearly defined previously, we computationally isolated the neural and immune cells for subsequent subcluster analysis. The immune cells were identified as macrophages (MΦ) based on their high expression of *MSR1* (Fig. 6G). Following the correction of ribosomal gene expression, reclustering yielded 12 subclusters labeled as M1-M12 (Fig. 6A, B). Among these, the M1, M2 and M4 subclusters were characterized as pro-inflammatory due to their high expression of *IL1B*, *HS3ST2* and *S100A8*, respectively. The M3 subcluster was identified as antigen-presenting cells based on their high expression of MHC-II molecules (*HLA-DQA1*, *HLA-DPB1*, and *HLA-DMB*). In contrast, the M6, M7, and M8 represented three anti-inflammatory subclusters due to their high expression of *CD52*, *IL1RN*, and *GREM1*, respectively. The identification of these subclusters suggested the presence of macrophage polarization. Surprisingly, two double-marker subclusters were identified based on the expression of stromal (M12, high *COL1A1*) or neural (M9, high *SOX2*) markers, in addition to immune genes (Fig. 6B, G). Recent research has reported the identification of stromal and immune double-positive [48], as well as neural and immune double-positive clusters [49–51]. However, it is important to exercise caution when considering these clusters due to the potential impact of remnants in other locations, which could affect the characterization of other cell types but may also reflect intimate interactions between the two cell types [52]. Additional macrophage subclusters were identified based on their highly expressed genes, including M5 (high *NMB*), M10 (high *GGCX*), and M11 (high *ABCG1*). Normal cells were found in small quantities in M2, M3, M5, M6, M7, M8, M10, and M11 subclusters, while they were absent in the pro-inflammatory (M1and M4) and MΦ-neural (M9) subclusters (Fig. 6 C). In contrast, cells from NTDs were generally elevated in all of the macrophage subclusters, especially in the SBA3 sample (Fig. 6 C). Regarding expression levels, a significantly higher expression of most marker genes was observed in normal cells compared to NTDs cells only in the M12 subcluster, while the other subclusters exhibited the opposite trend (Additional file 3: Fig. S3A). In the neural clusters, two subclusters were identified as neural glia (N7, high *S100B* and N8, high *CRYAB*), three as cycling (N1, high *CCNB2*, N6, high *HIST1H1D*, and N10, high *FAM83D*), and two as neuron subclusters (N2, high *NEFM* and N4, high *SLC7A11*) (Fig. 6 D, E). Additionally, four double-positive subclusters were identified, including neuromuscular (N9, high *ACTA2*), neurostromal (N3, high *DSP*, and N5, high *COL6A2*), and neuroimmune (N11, high *MSR1*) (Fig. 6 E, G). The emergence of these double-positive subclusters in neural cells may have the same underlying reason as in immune cells. Normal cells were found in N3, N5, N9, and N10 subclusters, but were absent in N1, N2, N6, N7, N4, N8, and N11 subclusters (Fig. 6F). On the other hand, cells from NTDs showed a significant increase in all neural subclusters, particularly in the SBA3 and SBA4 samples (Fig. 6F). Furthermore, normal cells displayed higher expression levels of most marker genes in N3 and N5 subclusters compared to NTDs cells, while cells in other subclusters showed the opposite trend (Additional file 3: Fig. S3B). These findings suggest that stromal signatures reflect cells shed during fetal development, while increased pro-inflammatory, neuron, and glia signatures indicate NTDs progression. Notably, two subclusters (M9 and N11) expressed both neural and immune markers, but cannot be categorized as typical microglia due to their absence of *P2RY12*, *TMEM119*, and *CX3CR1* (Additional file 3: Fig. S3D, E). However, these results may reflect close interactions between macrophages and neural cells, which could potentially contribute to neuroinflammation in NTDs.

### Significant macrophage polarization uncovered in NTDs

**Fig. 7:**
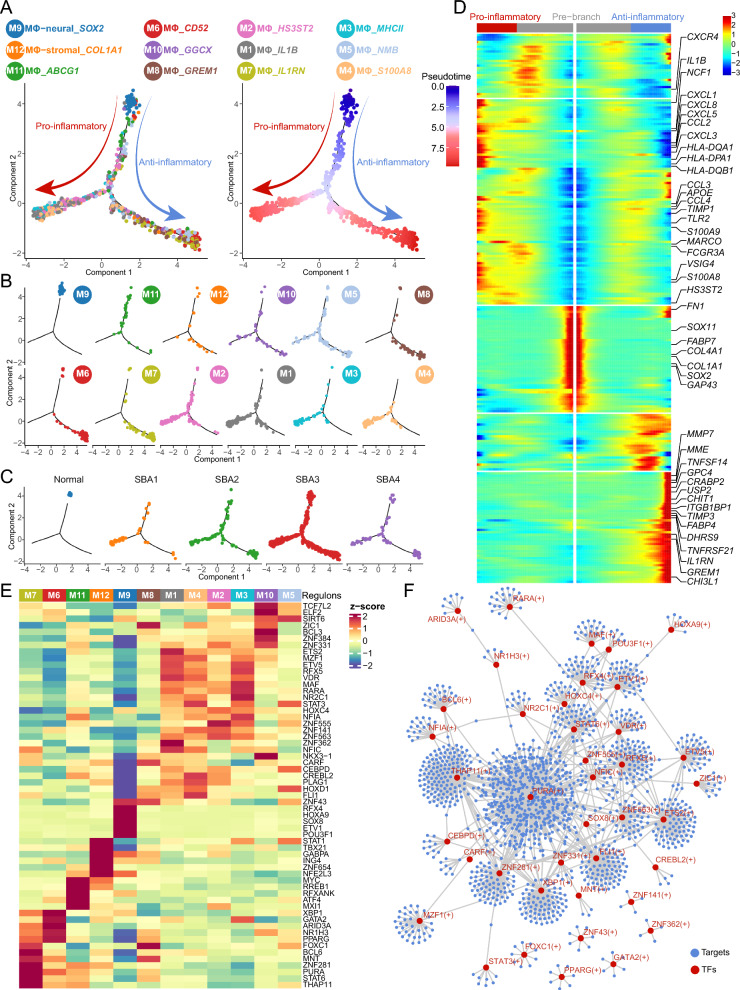
Cell trajectory and GRNs analysis for macrophage revealing distinct polarization in human NTDs. **A** Cell trajectory of macrophage displayed by subtypes (left) and pseudotime (right), respectively. **B** Each subtype exhibited separately along the cell trajectory of macrophage. **C** Cells from different samples projected to the macrophage trajectory. **D** Visualization of genes related to macrophage polarization state using branch heatmap. **E** Display of the top 5 regulons of average activity for each subtype using heatmap. **F** GRNs of the top 5 regulons in M1, M2, M3, M4, M6, M7, and M9 showing the TFs and target genes

The macrophage landscape in NTDs exhibited both anti-inflammatory and pro-inflammatory properties, suggesting the possible emergence of macrophage polarization. To further explore this process, we conducted cell trajectory analysis. Two distinct branches were clearly identified along the differentiation pseudotime (Fig. 7A). The first branch predominantly encompassed four subclusters: M1, M2, M3, and M4 (Fig. 7B). These subclusters exhibited the expression of well-known pro-inflammatory markers such as *IL1B*, *S100A8*, *TLR2*, and *CCL3*, along with a novel pro-inflammatory marker, *HS3ST2* [53] (Figs. 6B, 7D). In contrast, the second branch consisted of M6, M7, and M8 subclusters, which expressed known anti-inflammatory markers such as *CD52*, *IL1RN*, *GREM1* and *MMP7* (Figs. 6 B, 7B, D). Additionally, cells in subcluster M9 (expressing *SOX2*) were confined to the initial position, while subcluster M11 was distributed along the per-branch of the pseudotime trajectory (Fig. 7B). Notably, subclusters M5 (expressing *NMB*), M10 (expressing *GGCX*), and M12 (expressing *COL1A1*) were not restricted to any specific location (Fig. 7B). Focusing on M9, the positional pattern of this neuroimmune subcluster in the cell trajectory might imply that the initiation of macrophage activation was triggered by intimate interactions between neural and immune cells. The expression patterns of these subclusters exhibited divergent trends in the expression of pro- and anti-inflammatory genes, indicating distinct polarization of macrophages (Fig. 7D). The cells in NTDs were located along the entire cell trajectory, reflecting divergent activation properties (Fig. 7C). In contrast, cells in normal tissue predominantly gathered only at the initiation position of the cell trajectory, representing a resting state (Fig. 7C). Furthermore, we conducted GRNs analysis to assess the activities of regulons in each subclusters. A total of 216 regulons were identified in macrophages, and the average activities of regulon in each subcluster were calculated (Fig. 7E). The top five regulons for each subcluster further confirmed the polarization of macrophages, as indicated by the higher expression of anti-inflammatory TFs such as STAT6 and PPARG in M7 and M6, and pro-inflammatory TFs such as STAT3 and CEBPD in M1, M2, M3, and M4 (Fig. 7 E, F). Additionally, in the subclusters where normal cells were present, the Regulon specificity score (RSS) of top specific regulons (z-score > 2) in NTDs cells was higher compared to normal cells, except for the M12 subcluster (Additional file 3: Fig. S3C). These findings collectively confirm the distinct properties of normal and NTDs cells in terms of macrophage polarization.

### Revealing cell communications between macrophages and neural cells in NTDs

**Fig. 8:**
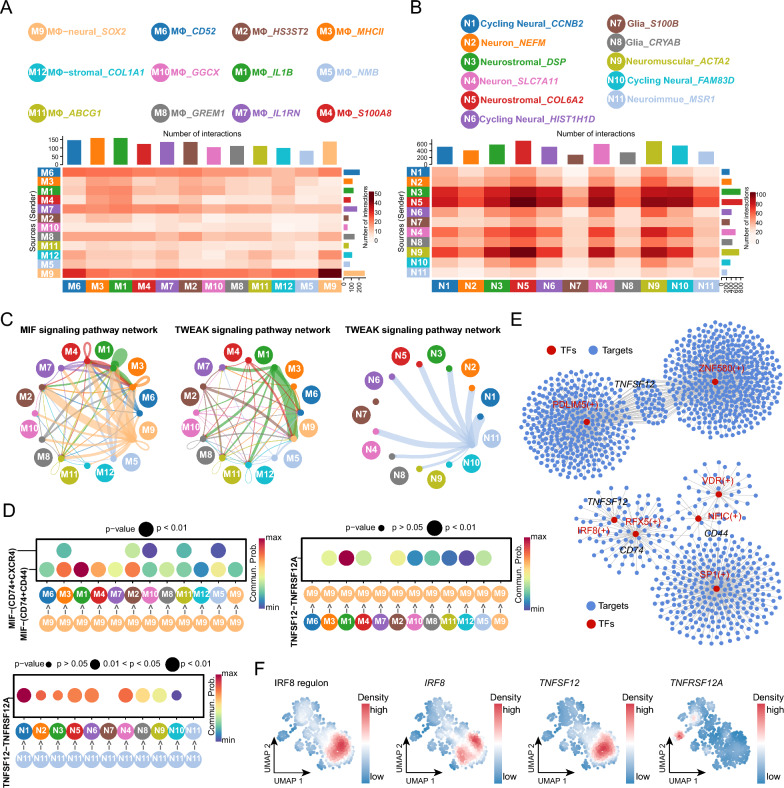
Cell communication of macrophage and neural cells in human NTDs. **A**, **B** Construction of cell communication based on the number of interactions between macrophage and neural cells, respectively. **C** Network of MIF and TWEAK signaling pathways in macrophage and neural cells. **D** Detailed ligand-receptor pairs of MIF and TWEAK signaling pathways in macrophage and neural cells displayed in bubble plots. **E** GRNs of MIF and TWEAK signaling pathways in macrophage and neural cells. **F** Density plots of IRF8 regulon, *IRF8*, *TNFSF12*, and *TNFRSF12A* in macrophage

To further investigate the neuroimmune characteristics, the cell communication analysis was conducted. The numbers of interactions between macrophages and neural cells were assessed to construct their respective cell communication networks (Fig. 8A, B). Notably, subcluster M9 exhibited a high level of interactions with other cell types (Fig. 8A). This observation led to the identification of two signaling pathways, stemming from the intense interaction between neuroimmune cells and other subclusters (Fig. 8C). In the MIF signaling pathway, subcluster M9, serving as a source, effectively targeted subclusters M1, M2, M3 and M4. Conversely, in the TWEAK signaling pathway, subcluster M9, acting as a receptor, was prominently targeted by subcluster M1 (Fig. 8 C). These two pathways have been reported to exhibit pro-inflammatory signaling [54, 55]. Additionally, subcluster N10, as a source, targeted all neural subclusters except for N7 in TWEAK signaling pathway. These interactions were predicated on the receptor-ligand pairs of MIF-(CD74 + CD44), MIF-(CD74 + CXCR4), and TNFSF12-TNFRSF12A (Fig. 8D). Combined with the GRN analysis, *TNFSF12* was a downstream gene regulated by PDLIM5 and ZNF580 in N11-interacted subclusters, and was regulated by IRF8 in M9-interacted subclusters (Fig. 8 E). Interestingly, IRF8 also emerged as a key TF revealed in the bulk transcriptome and methylome of the brain and spinal cord (Fig. 3B, C, Fig. 5B). Furthermore, IRF8 is suppressed by STAT5, an anti-inflammatory TF, and conversely promoted by STAT1, a pro-inflammatory TF [44]. The gene and regulon expression of IRF8 and its target *TNFSF12* were enriched in pro-inflammatory subclusters, while *TNFRSF12A*, as the target of *TNFSF12*, was enriched in neuroimmune subclusters (Fig. 8 F), thus forming a comprehensive regulatory network. These observations suggest that the TWEAK signaling pathway may play an important role in mediating pro-inflammatory cell communication between macrophages and neural cells in NTDs.

### Potential prenatal diagnostic markers unveiled in NTDs through integrating bulk- and scRNA-seq transcriptome

**Fig. 9:**
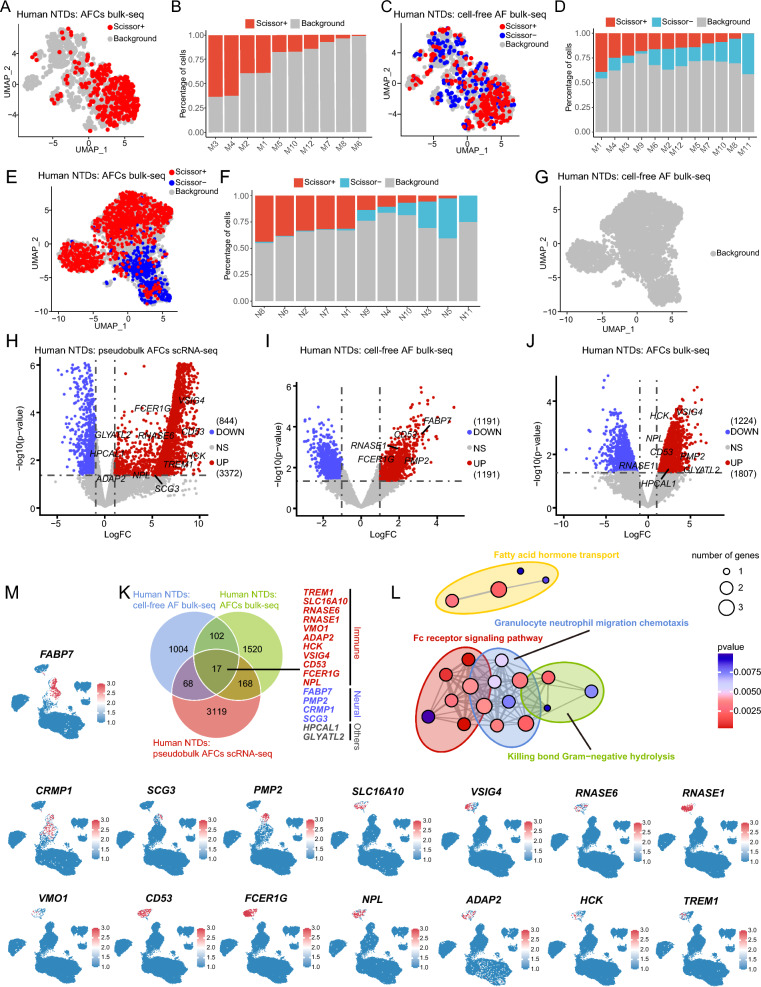
Integrated human bulk- and scRNA-seq data revealing potential prenatal diagnostic markers of NTDs. **A** Integration of bulk-seq data of human primary AFCs with scRNA-seq data of human macrophages. The Scissor + designation represents a positive association with NTDs, while Scissor- signifies the opposite. Cells with no identified correlation were defined as Background. **B** Proportion of human primary AFCs-integrated cells with different correlations in each phenotypically related subtype of macrophages. **C** Integration of bulk-seq data of human cell-free AF with scRNA-seq data of human macrophages. **D** Proportion of human cell-free AF- integrated cells with different correlations in each phenotypically related subtype of macrophages. **E** Integration of bulk-seq data of human primary AFCs with scRNA-seq data of human neural cells. **F** Proportion of human primary AFCs-integrated cells with different correlations in each phenotypically related subtype of human neural cells. **G** Integration of bulk-seq data of human cell-free AF with scRNA-seq data of human neural cells. **H** Pseudobulk DEGs analysis of scRNA-seq data from cultured human AFCs. **I** DEGs analysis of bulk-seq data from human cell-free AF. **J** DEGs analysis of bulk-seq data from human primary AFCs. **K** Venn diagram displaying DEGs shared in different contexts of human AF. **L** GO enrichment analysis of 15 candidate genes for prenatal diagnosis. **M** Expression patterns of the 15 candidate genes for prenatal diagnosis in human AFCs at single-cell resolution

Since we have identified neuroinflammation as a prominent pathological feature of NTDs, we were interested in determining whether this feature could serve as a reliable biomarker for the prenatal diagnosis of NTDs through AF examination. First, to address potential differences between cultured cells and primary cells, we integrated bulk-seq data from human primary amniotic fluid cells (AFCs, GEO, GSE4182, 4 NTDs vs. 5 normal controls) [56], as well as human cell-free AF (GEO, GSE101141, 10 NTDs vs. 10 normal controls) [57], with scRNA-seq data from cultured human AFCs (GEO, GSE206696, 4 NTDs vs. 1 normal control) [13]. This integration allowed us to identify common characteristics present in NTDs. Our analysis of bulk-seq data from primary AFCs and cell-free AF, as well as CNS tissues and cultured AFCs, revealed a significant increase in immunological signatures (Additional file 4: Fig. S4A, B). In contrast, neural features such as *FEZ1*, *GAP43*, *FABP4* and *PTPRZ1* were found to be elevated in AF compared to those that were downregulated in CNS tissues (Additional file 4: Fig. S4A, B, Fig. 1E, H). By integrating bulk-seq AFCs and AF data with single-cell macrophage data, we discovered that cells positively associated with NTDs (Scissor +) shared common signatures enriched in pro-inflammatory subclusters, including M1, M2, M3, and M4 (Fig. 9A–D). Furthermore, upon integrating bulk-seq AFCs with single-cell neural cells, we observed that Scissor + cells were highly enriched in N1, N2, N6, N7, and N8, but not in double-positive cells, which exhibited an accumulation of Scissor- cells (negatively related to NTDs) (Fig. 9E, F). However, no phenotypically related cells were identified when integrating bulk-seq AF with single-cell neural cells (Fig. 9G). The common pattern of AF across different contexts allowed for the identification of shared prenatal diagnostic markers. It is generally recognized that prenatal diagnostic markers should be detectable in a cost-effective and straightforward manner at the bulk level, rather than through complex and expensive single-cell analysis. Therefore, we conducted pseudobulk-seq DEGs analysis using the scRNA-seq data from cultured AFCs (GEO, GSE206696, 4 NTDs vs. 1 normal control) and performed routine DEGs analysis using two bulk-seq datasets from AF (GEO, GSE101141, 10 NTDs vs. 10 normal controls) and AFCs (GEO, GSE4182, 4 NTDs vs. 5 normal controls) (Fig. 9H–J). A total of 17 genes were found to be upregulated in all three analysis results, comprising 11 immune genes (*TREM1*, *SLC16A10*, *RNASE6*, *RNASE1*, *VMO1*, *ADAP2*, *HCK*, *VSIG4*, *CD53*, *FCER1G*, and *NPL*), four neural genes (*FABP7*, *PMP2*, *CRMP1*, and *SCG3*), and two other genes (*HPCAL1* and *GLYATL2*) (Fig. 9 K). These genes were enriched in immune or fatty acid related process (Fig. 9 L), consistent with results in CNS mentioned above (Fig. 2) These immune and neural genes were expressed in immune or neural clusters at the single-cell level of AFCs (Additional file 2: Fig. S2A, Fig. 9M). As anticipated, the immune genes showed upregulation in the transcriptomic data of brain and spinal cord with NTDs, while the neural genes displayed downregulation (Additional file 4: Fig. S4C, D). Subsequently, these 15 genes were identified as candidate prenatal diagnostic markers for further analysis.

### Identifying the conserved prenatal diagnostic markers indicating the severity of NTDs

**Fig. 10:**
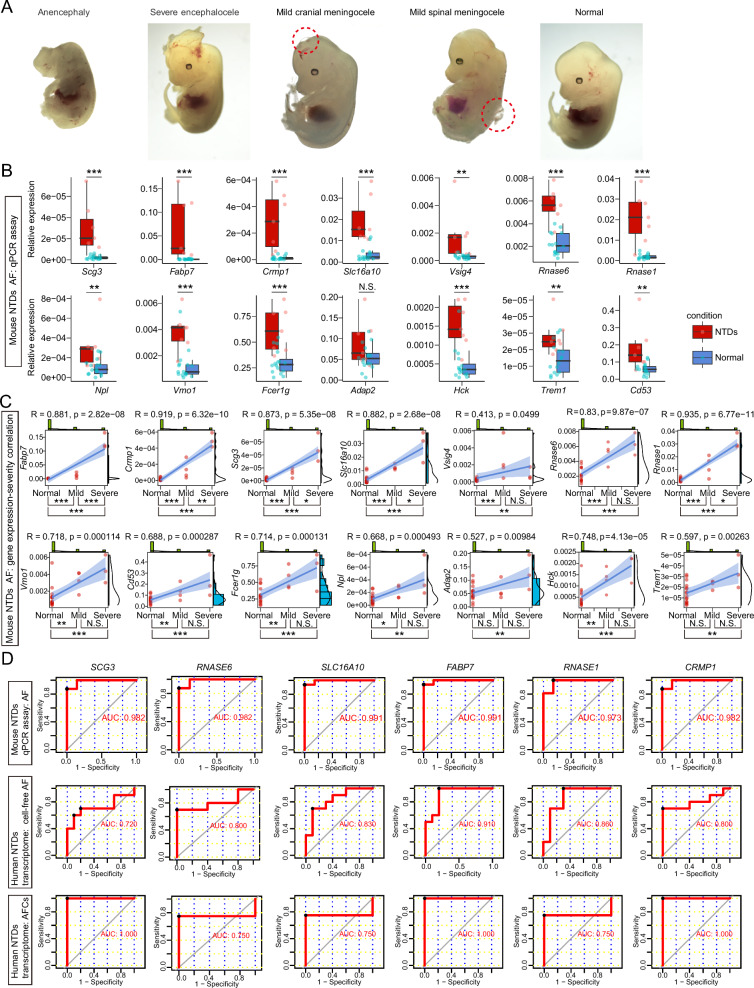
Identification of conserved prenatal diagnostic markers indicating the severity of NTDs. **A** Assessment of RA-induced NTDs mouse models, exhibiting distinct lesions in the brain or spinal cord. **B** Relative expression levels of candidate genes for prenatal diagnosis in these mouse models detected by qPCR assay. **C** Performance of correlation analysis between gene expression (qPCR detected) and the severity of NTDs in mouse models, with R representing the coefficient value. **D** Application of ROC analysis for prenatal diagnostic genes in the RA-induced mouse model (qPCR detected) and human transcriptome. *P < 0.05; **P < 0.01; ***P < 0.001, non-significant, N.S

Retinoic acid (RA), an active derivative of vitamin A, plays a critical role in neural system development [58]. Imbalances in RA levels, either deficiency or excess, can contribute to certain types of NTDs [59]. Macrophages, being target cells for RA, express retinoic acid receptors (RARs) and produce inflammatory cytokines, which are inhibited by RA [60]. Consistently, we observed that *RARA* (Retinoic Acid Receptor Alpha) was highly expressed in pro-inflammatory macrophage subclusters, including M1, M2, M3, and M4 (Fig. 7E). Furthermore, we detected dysregulation of several genes related to the RA signaling pathway in human NTDs, including *RAI1*, *RARRES1*, *RARRES3*, *CYP26B1*, *RARB*, *THRB*, *RARG*, *RARA*, *RXRA*, *THRA*, *BRINP3*, and *PPARG* (Additional file 5: Fig. S5). This suggests a close relationship between the human samples used in our study and RA metabolism. Therefore, we subsequently established a mouse model of RA-induced NTDs [17] to identify conserved markers among prenatal diagnostic candidates. The incidence of NTDs caused by maternal RA overdose was 30.43% in our study (7 out of 23 embryos). Our study identified four subtypes of NTDs: anencephaly (severe), severe encephalocele, mild cranial meningocele, and mild spinal meningocele, each demonstrating diverse lesions in the brain or spinal cord compared to normal conditions (Fig. 10 A). AF was extracted from the E13.5 embryos, followed by gene expression analysis with a qPCR assay. Initially, we evaluated the expression of the key inflammatory TFs (*STAT3*, *STAT1*, *STAT2*, *NFKB1*, *IRF1*, *IRF8*, *HDAC1*, *EGR2*, *IRF4*, and *STAT6*) mentioned above in AF, and most of them exhibited higher expression in mice with NTDs (Additional file 6: Fig. S6). The presence of neural gene expression in AF suggests abnormal leakage from the nervous system. Consequently, we investigated the gene expression of the four neural genes (*FABP7*, *SCG3*, *CRMP1*, and *PMP2*) in both the developing brain and spinal cord across mouse and human (Additional file 7: Fig. S7, Additional file 8: Fig. S8). Our analysis revealed that *PMP2* was nearly absent in the developing mouse brain (Mouse Brain Atlas, http://mousebrain.org/development/) [61] and spinal cord (ArrayExpress, E-MTAB-7320) [62] (Additional file 7: Fig. S7), while it was highly expressed in the developing human brain (GEO, GSE120046) [63] and spinal cord (GEO, GSE136719) [64] (Additional file 8: Fig. S8). Therefore, *PMP2* was excluded from further analysis. It is worth noting that the CNS is known to host various immune cells, particularly microglia, under normal conditions, indicating the potential presence of candidate immune markers within the CNS (Additional file 7: Fig. S7, Additional file 8: Fig. S8). Through qPCR assay, we discovered that the four subtypes of NTDs had specific gene expression patterns associated with their severity (Additional file 9: Fig. S9). In the two main groups (NTDs vs. Normal), the expression levels of all candidate genes, except *Adap2*, were significantly higher than normal in RA-induced mouse NTDs detected by qPCR assay. (Fig. 10B). Subsequently, we conducted an analysis to examine the correlation between gene expression and the severity of NTDs. This analysis revealed a positive correlation between the expression levels of all 14 genes and the severity of NTDs. Among them, *Fabp7*, *Crmp1*, *Scg3*, *Slc16a10*, *Rnase6*, and *Rnase1* stood out as the strongest candidates due to their coefficient (R) exceeding 0.8 (Fig. 10C). These genes were selected for Receiver Operating Characteristic (ROC) analysis to assess their diagnostic capabilities. The results indicated that these six genes held diagnostic value, as evidenced by their Area Under the Curve (AUC) exceeding 0.5 in both mouse (qPCR assay) and human (transcriptome data) (Fig. 10D). Therefore, *FABP7*, *CRMP1*, *SCG3*, *SLC16A10*, *RNASE6*, and *RNASE1* may serve as potential prenatal diagnostic markers, indicating the severity of NTDs. The approach, which we term the “M + N” (macrophage + neural) method, has the potential to provide valuable insights into the diagnosis of NTDs.

## Discussion

By integrating data of multiomics, the findings of this study provide valuable insights into the interplay of neuroinflammation and NTDs within the context of the CNS and AF. Our results uncover the cellular heterogeneity, gene regulatory networks, and pinpoint several pivotal genes and pathways that are instrumental for comprehending and conducting prenatal diagnosis of NTDs.

Understanding the pathological characteristics of NTDs is crucial for guiding research into prevention strategies, prenatal diagnosis, and potential interventions aimed at minimizing the impact of these debilitating congenital malformations. We found that neuroinflammation, a complex process involving the activation of various immune cells within the central nervous system, is an important pathological feature of NTDs. Among these immune cells, microglia, the resident macrophages of the CNS, play a pivotal role in orchestrating the inflammatory response [65]. Emerging evidence suggests that microglia are not only involved in the maintenance of normal CNS function, but also participate in the pathogenesis of neuroinflammatory diseases, including multiple sclerosis [66], Alzheimer's disease [67], and Parkinson's disease [68]. However, due to the lack of single-cell resolved atlas of CNS tissues, it remains unclear whether microglia are involved in the pathogenesis of the neuroinflammatory condition in human NTDs. In a rat model of RA-induced NTDs, activated microglia were observed in the exposed neural spinal cord [69]. Our findings indicated an absence of microglial signature in cultured AFCs from NTDs, failing to accurately reflect the actual condition of the CNS and primary AFCs. Thus, future research efforts should aim to elucidate the precise cell heterogeneity and key regulatory pathways of CNS and primary AFCs in NTDs at single-cell resolution. Furthermore, as the human samples we analyzed were only from the second trimester (gestational week 13–27) and not during the critical period of neural tube closure (gestational week 3–4), our study's findings regarding the role of neuroinflammation in understanding the pathogenesis of NTDs are quite limited. Therefore, more research is needed to establish the temporal relationship and underlying mechanisms between NTDs and neuroinflammation.

The AF, a crucial component of the intrauterine environment, is known to harbor a diverse population of immune cells that play a pivotal role in maintaining maternal–fetal tolerance and protecting against intrauterine infections [70]. Among the various immune cells present in the AF, macrophages, neutrophils, T cells, B cells, and natural killer cells have been identified as key players in orchestrating immune responses within the gestational compartment [71–73]. However, we only identified macrophage in cultured AFCs from fetuses with NTDs. This could possibly be cell competition in culture [74] or the “drop-out” phenomenon in scRNA-seq [75]. Macrophages play a crucial role in the immune system and can be polarized into distinct phenotypes in response to different microenvironmental signals [76]. Traditionally, macrophage polarization is categorized into two main phenotypes. One is classically activated and involved in pro-inflammatory responses, while the other is alternatively activated and contribute to tissue repair and anti-inflammatory processes [77]. In this process, the JAK-STAT signaling pathway has been reported to play important roles [78], which aligns with our results (Fig. 7 E, F). The relationship between macrophage polarization and NTDs is still not fully understood. One hypothesis suggests that fetal-origin anti-inflammatory macrophages might have a significant impact on the development and closure of the neural tube. Generally, factors that prevent NTDs tend to enhance the activity of anti-inflammatory macrophages, while teratogenic factors are likely to influence macrophage polarization toward pro-inflammatory macrophages and away from anti-inflammatory macrophages [79]. In a mouse model, it has been reported that maternal diabetes exacerbates inflammation induced by teratogens, leading to NTDs accompanied by increased activity of amoeboid microglia/brain macrophages (activated) and elevated expression of pro-inflammatory cytokines [80]. Our findings highlighted the diverse phenotypic states of macrophage in NTDs, ranging from a surveillant phenotype under homeostatic conditions to an activated, pro- or anti- inflammatory state in response to neural lesion, thereby contributing to the neuroinflammation of NTDs.

We found that TWEAK signaling (TNFSF12-TNFRSF12A) plays a significant role in NTDs by facilitating pro-inflammatory cell communication between macrophages and neural cells. Tumor necrosis factor (TNF)-like weak inducer of apoptosis (TWEAK, encoded by *TNFSF12*) is a member of the TNF superfamily. When it binds to fibroblast growth factor-inducible 14 (Fn14, encoded by *TNFSF12A*), the TWEAK/Fn14 pathway can activate both canonical and noncanonical NF-kappa B signaling pathways, which regulate cellular apoptosis and inflammation [81]. In the context of multiple sclerosis, the presence of TWEAK-expressing macrophages/microglia in cortical lesions and inflamed leptomeninges, along with extensive myelin loss, astrocytosis, neuronal damage, and vascular abnormalities, supports the possibility that TWEAK signaling-mediated macrophages/microglia may have potentially detrimental effects on neural cells expressing the Fn14 (TNFSF12A) receptor [82].

AF examination is a common method for prenatal diagnosis of potential genetic disorders or congenital abnormalities [83, 84]. Focusing on NTDs, the existing prenatal diagnostic markers such as acetylcholinesterase and alpha-fetoprotein have limitations in terms of sensitivity, specificity, and technical requirements [23, 85]. We identified a set of six novel markers, including *FABP7*, *CRMP1*, *SCG3*, *SLC16A10*, *RNASE6*, and *RNASE1*, for the prenatal diagnosis of NTDs across a spectrum of severity. Surprisingly, these genes have not been previously reported in relation to either human patients or animal models with NTDs. Among them, nervous system-related genes, including *FABP7*, *CRMP1*, and *SCG3*, play important roles in neural development, neurodegenerative disorders, and neuroendocrine systems [86–90]. On the other hand, immune system-related genes, including *SLC16A10*, *RNASE1*, and *RNASE6*, function in immune responses, antimicrobial activity, and enhancing anti-tumor immunity [91–94]. And these genes were highly expressed in macrophage or neural clusters at the single-cell level of AFCs. (Additional file 2: Fig. S2A, Fig. 9M). Therefore, we termed this approach as the "M + N" (Macrophage + Neural) method. The double type markers may enable high specificity in prenatal diagnosis of NTDs. For example, it has been reported that the number of macrophages in AF was increased in the presence of intra-amniotic infection [70] and spina bifida [95]. Thus, our “M + N” method may exhibit more specificity due to the ability to exclude nonspecific results from other inflammatory conditions with no neural signatures such as intra-amniotic infection. This diagnosis tool should be further validated in larger human cohorts to translate our findings into clinical application.

## Conclusions

In conclusion, our study underscores the importance of neuroinflammation in the progress of NTDs and highlights the potential of specific inflammatory and neural markers as novel diagnostic tools. Our findings pave the way for future research aimed at elucidating the mechanisms linking neuroinflammation and NTDs, and developing effective strategies for early detection and prevention of these congenital disorders.

### Supplementary Information

**Additional file 1: Figure S1.** Schematic diagram of data analysis and experiments validation in this study. NTDs, neural tube defects; CNS, central nervous system; RA, retinoic acid; GW, gestational week.

**Additional file 2: Figure S2.** The single-cell atlas of cultured AFCs from human fetuses with NTDs. **A** UMAP plot of cultured human AFCs grouped by annotated cell types. RETCs: renal tubular epithelial cells; NnPCs: nephron progenitor cells; IM: immune cells; SCT: syncytiotrophoblasts. **B** Proportion of cells from different samples in each type of cultured human AFCs, with SBA representing Spina Bifida. **C** Projection of cells from different samples onto the UMAP plot of cultured human AFCs. **D** Dotplot displaying the top five markers of each cell type in cultured human AFCs.

**Additional file 3: Figure S3.** Expression patterns of genes and regulons in macrophages and neural cells of human NTDs. **A**, **B** Expression levels of the top marker genes in macrophage and neural subtypes from human NTDs and normal cells. **C** Expression levels of regulons with the top RSS in macrophage subtypes from human NTDs and normal cells. **D**, **E** Expression patterns of microglia markers in macrophage and neural cells of human NTDs, respectively, at single-cell resolution.

**Additional file 4: Figure S4.** DEGs analysis of human AF transcriptome from different contexts and the expression patterns of candidate genes for prenatal diagnosis in human CNS transcriptome with NTDs. **A**, **B** Heatmap visualization of DEGs in human cell-free AF and AFCs transcriptome, respectively, with the top DEGs displayed on the left and functional enrichment on the right. **C**, **D** Expression levels of candidate genes for prenatal diagnosis in human NTDs transcriptome of brain and spinal cord, respectively.

**Additional file 5: Figure S5.** Dysregulated expression patterns of RA signaling pathway related genes in human NTDs transcriptome and methylome. *P < 0.05; **P < 0.01; ***P < 0.001

**Additional file 6: Figure S6.** Expression patterns of pro-inflammatory TFs in RA-induced mouse NTDs amniotic fluid were detected by qPCR assay. *P < 0.05; **P < 0.01, non-significant, N.S.

**Additional file 7: Figure S7.** Expression patterns of candidate genes for prenatal diagnosis in developing mouse brain and spinal cord at single-cell resolution. **A** Expression patterns of candidate genes for prenatal diagnosis in the developing mouse brain. VLMCs: vascular leptomeningeal cells; VSM: vascular smooth muscle. OPCs: oligodendrocyte progenitors; EPEND: ependymal. **B** Expression patterns of candidate genes for prenatal diagnosis in the developing mouse spinal cord. NPCs: neural progenitor cells.

**Additional file 8: Figure S8.** Expression patterns of candidate genes for prenatal diagnosis in developing human brain and spinal cord at single-cell resolution. **A** Expression patterns of candidate genes for prenatal diagnosis in the developing human brain. EX: excitatory neuron; CR: Cajal-Retzius cells; IN: inhibitory neuron; Pons-neu: projection neuron in pons; Oligo: oligodendrocytes; Astro: astrocytes; MG: microglia; MΦ: macrophage; SMCs: smooth muscle cells; VECs: vascular endothelial cells; VLMCs: vascular leptomeningeal cells; DCs: Dendritic cells; RBCs: red blood cells. **B** Expression patterns of candidate genes for prenatal diagnosis in the developing human spinal cord. OPCs: oligodendrocyte progenitors.

**Additional file 9: Figure S9.** Expression patterns of candidate prenatal diagnostic markers in four subtypes of the RA-induced mouse NTDs model were examined by qPCR assay. *P < 0.05; **P < 0.01; ***P < 0.001, non-significant, N.S.

**Additional file 10: Table S1.** Primers used for qPCR assay.

## Data Availability

Public data that were re-analyzed here are from GEO with accession number GSE33111 (Expression array in CNS tissues of NTDs) (https://www.ncbi.nlm.nih.gov/geo/query/acc.cgi?acc=GSE33111), GSE69502 (Methylation array in CNS tissues of NTDs) [47], GSE101141 (Expression array in AF of NTDs) [57], GSE4182 (Expression array in AFCs of NTDs) [56], GSE120046 (scRNA-seq in developing human brain) [63], GSE136719 (scRNA-seq in developing human spinal cord) [64], GSE206696 (scRNA-seq in AFCs of NTDs) [13]; ArrayExpress with accession number E-MTAB-7320 (scRNA-seq in developing mouse spinal cord) [62]; Mouse Brain Atlas (http://mousebrain.org/development/) [61]

## Abbreviations

NTDs: Neural tube defects
AFCs: Amniotic fluid cells
AF: Amniotic fluid
CNS: Central nervous system
qPCR: Real-time quantitative polymerase chain reaction
GEO: Gene Expression Omnibus
RA: Retinoic acid
DEGs: Differentially expressed genes
FC: Fold change
PPI: Protein–protein interaction
KEGG: Kyoto Encyclopedia of Genes and Genomes
GO: Gene Ontology
GSEA: Gene Set Enrichment Analysis
GRNs: Gene regulatory networks
SEM: Mean ± standard error of the mean
ANOVA: Analysis of variance
N.S.: Non-significant
scRNA-seq: Single-cell RNA sequencing
GRNs: Gene regulatory networks
DMRs: Differentially DNA methylated regions
TFs: Transcription factors
MΦ: Macrophage
UMAP: Uniform Manifold Approximation and Projection
PCA: Principal component analysis
RSS: Regulon specificity score
ROC: Receiver Operating Characteristic
AUC: Area Under the Curve
GW: Gestational week
