# Supplementary figures and images for: Characterizing neuroinflammation and identifying prenatal diagnostic markers for neural tube defects through integrated multi-omics analysis
Source: J Transl Med. 2024 Mar 9;22:257. doi: 10.1186/s12967-024-05051-8 (PMC10924416; doi:10.1186/s12967-024-05051-8)

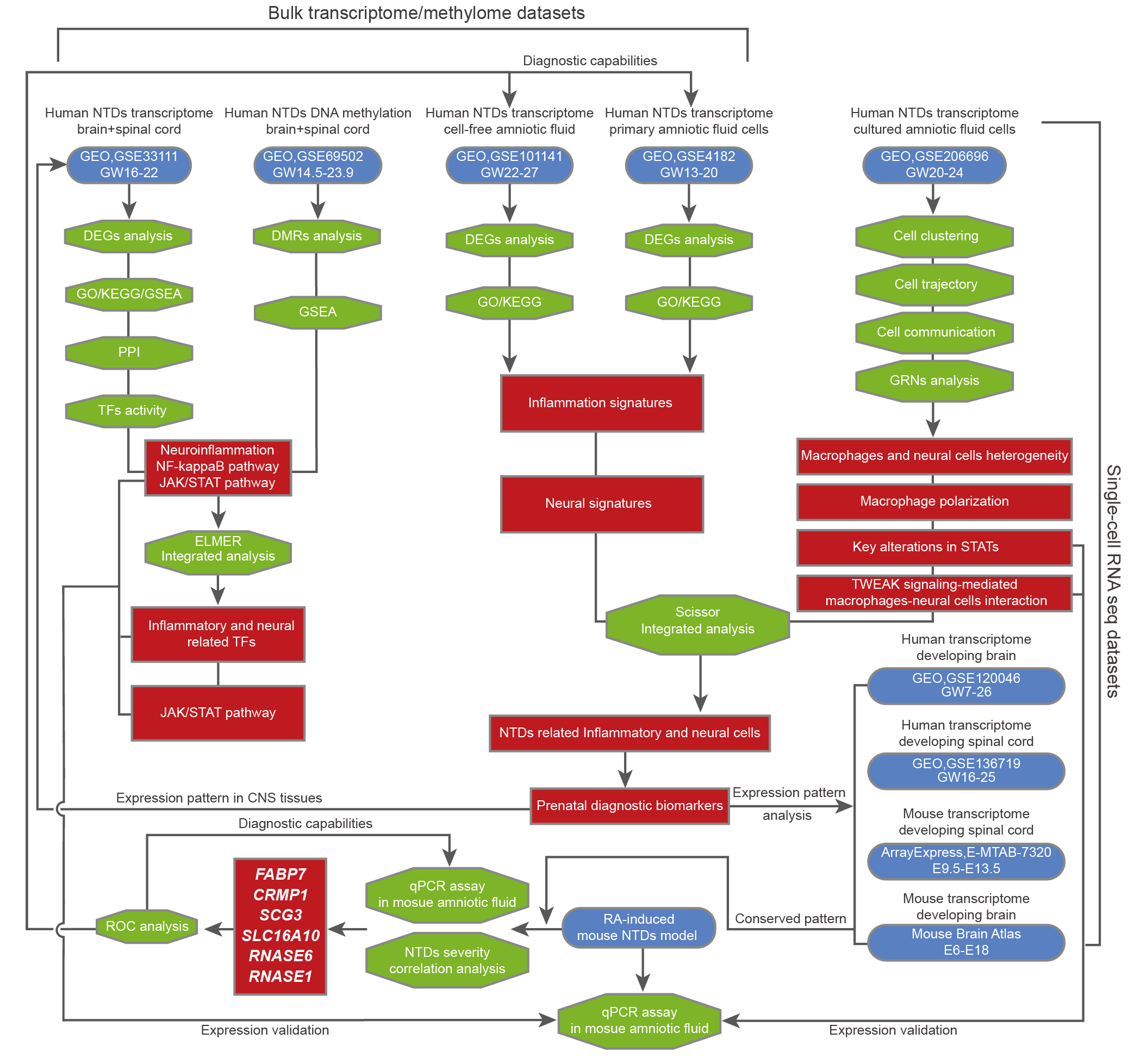

Supplement: Supplementary file 1 — Additional file 1: Figure S1. Schematic diagram of data analysis and experiments validation in this study. NTDs, neural tube defects; CNS, central nervous system; RA, retinoic acid; GW, gestational week. [file 12967_2024_5051_MOESM1_ESM.jpg]

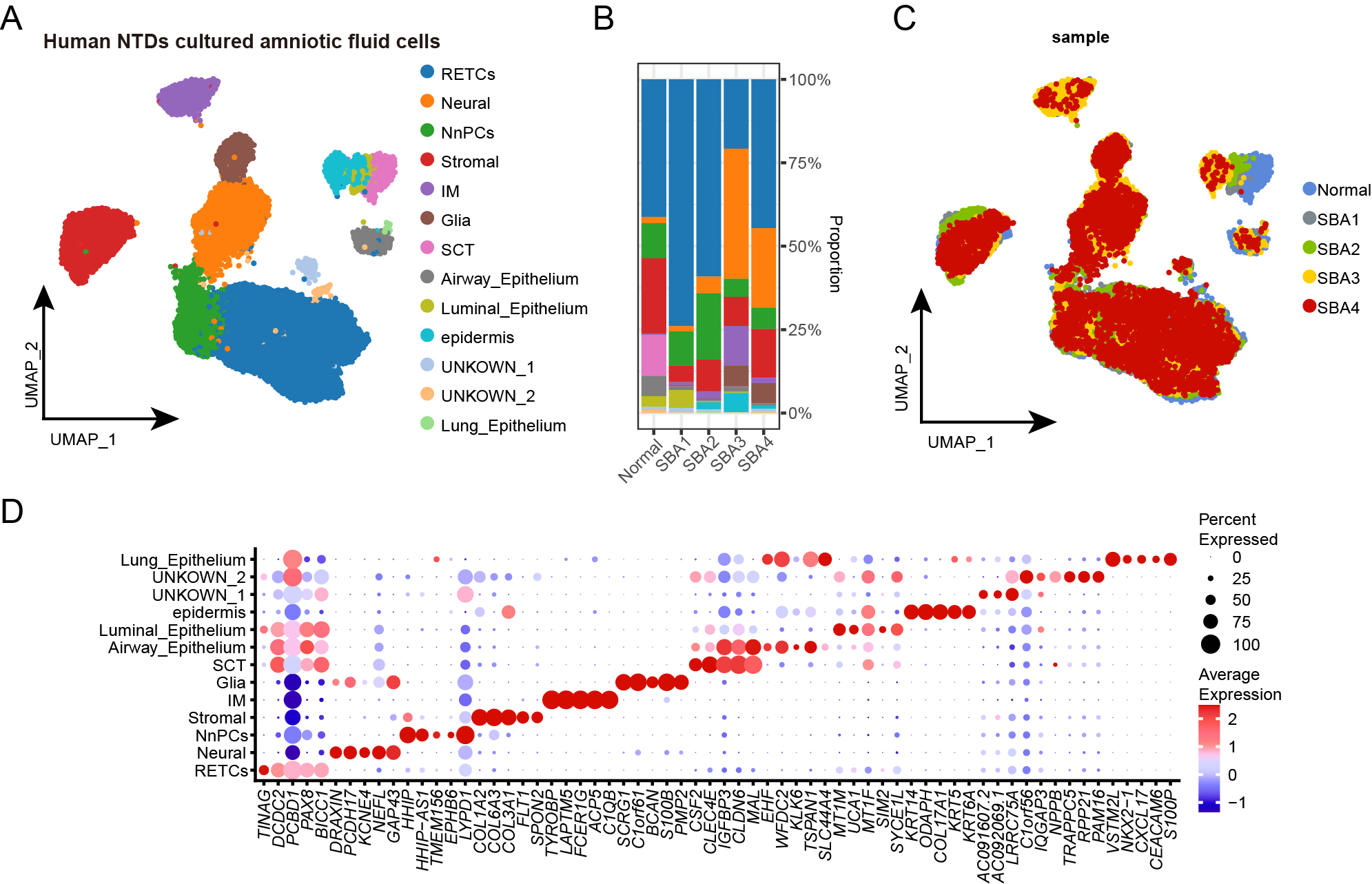

Supplement: Supplementary file 2 — Additional file 2: Figure S2. The single-cell atlas of cultured AFCs from human fetuses with NTDs. A UMAP plot of cultured human AFCs grouped by annotated cell types. RETCs: renal tubular epithelial cells; NnPCs: nephron progenitor cells; IM: immune cells; SCT: syncytiotrophoblasts. B Proportion of cells from different samples in each type of cultured human AFCs, with SBA representing Spina Bifida. C Projection of cells from different samples onto the UMAP plot of cultured human AFCs. D Dotplot displaying the top five markers of each cell type in cultured human AFCs. [file 12967_2024_5051_MOESM2_ESM.jpg]

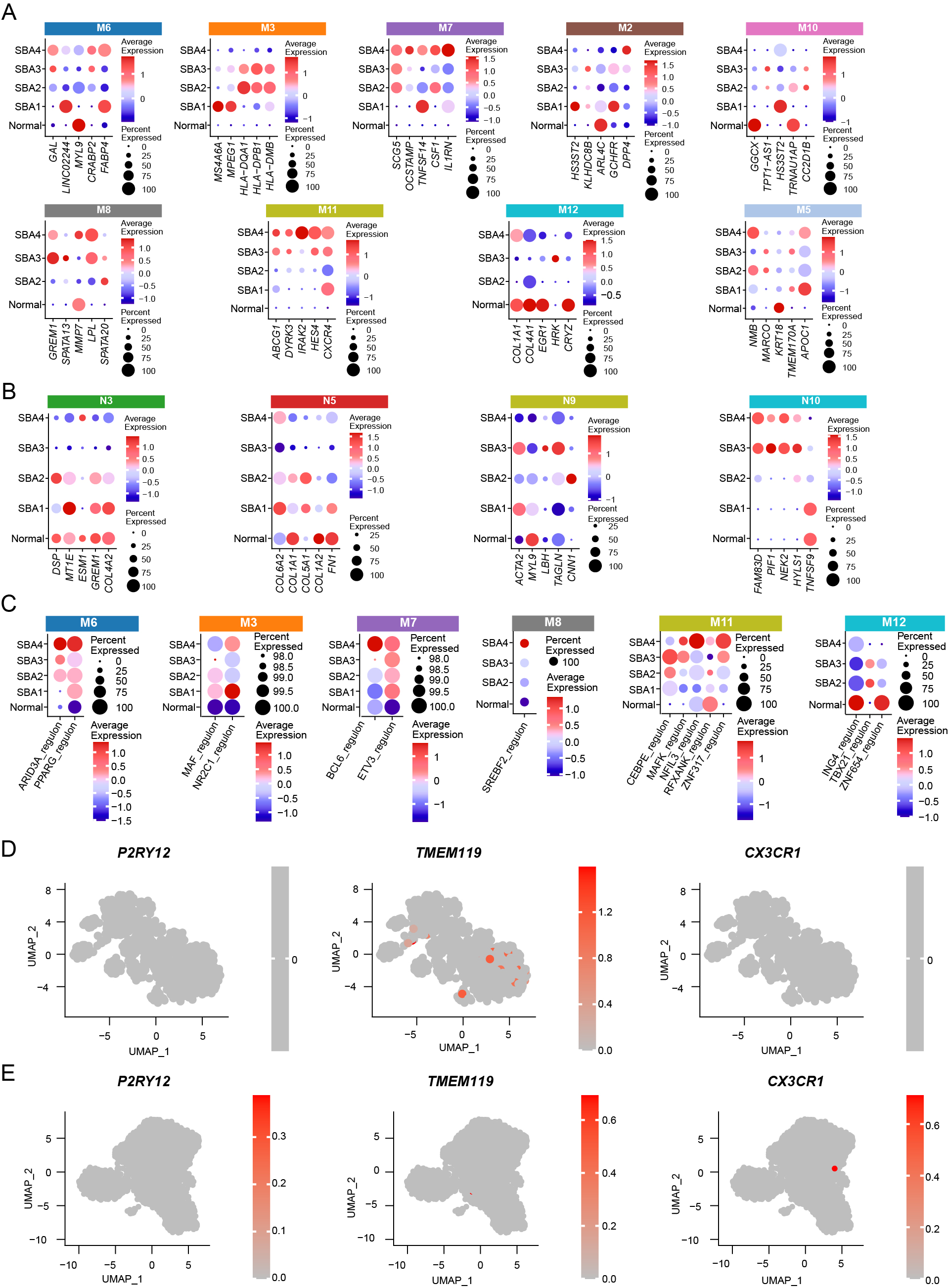

Supplement: Supplementary file 3 — Additional file 3: Figure S3. Expression patterns of genes and regulons in macrophages and neural cells of human NTDs. A, B Expression levels of the top marker genes in macrophage and neural subtypes from human NTDs and normal cells. C Expression levels of regulons with the top RSS in macrophage subtypes from human NTDs and normal cells. D, E Expression patterns of microglia markers in macrophage and neural cells of human NTDs, respectively, at single-cell resolution. [file 12967_2024_5051_MOESM3_ESM.jpg]

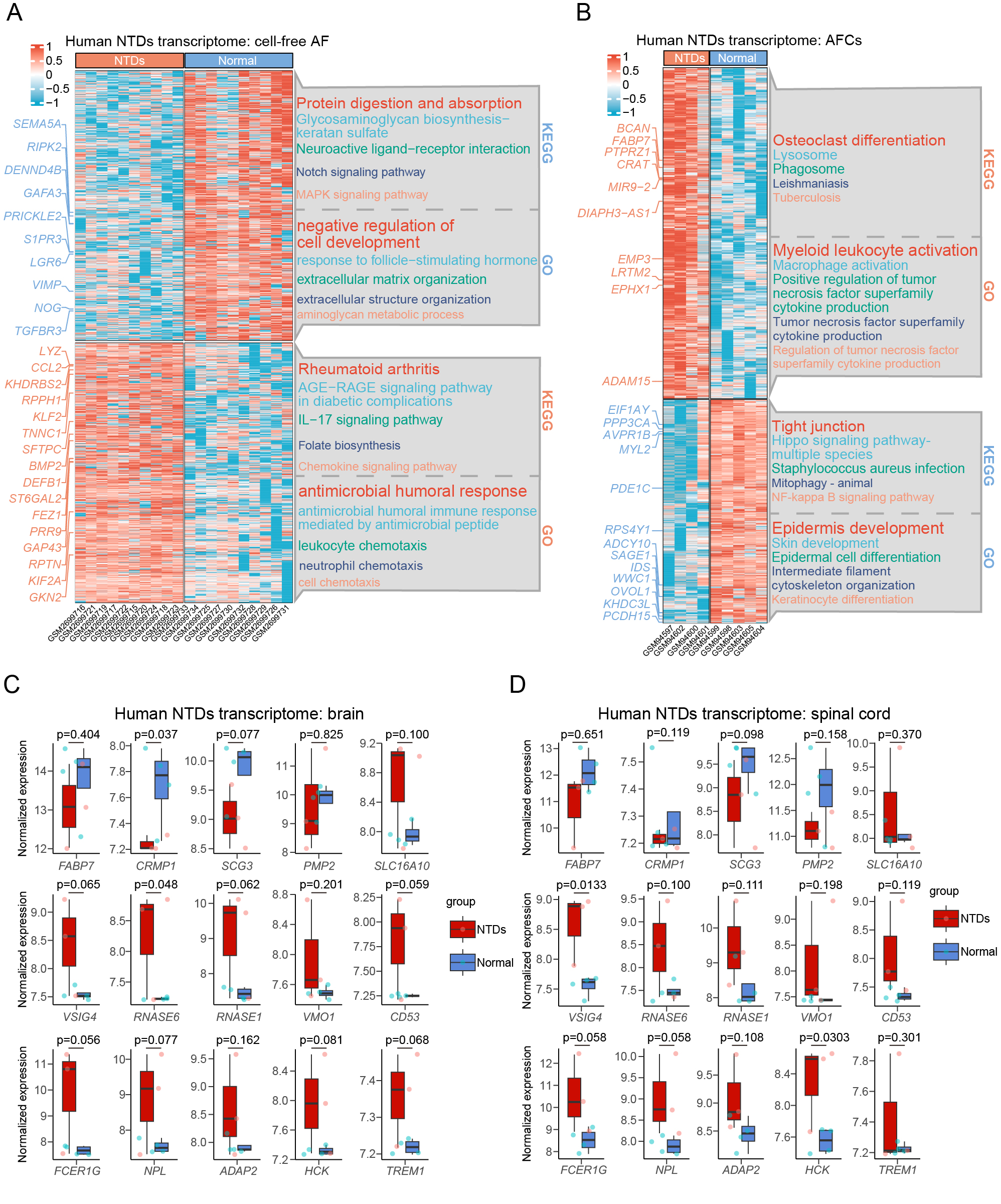

Supplement: Supplementary file 4 — Additional file 4: Figure S4. DEGs analysis of human AF transcriptome from different contexts and the expression patterns of candidate genes for prenatal diagnosis in human CNS transcriptome with NTDs. A, B Heatmap visualization of DEGs in human cell-free AF and AFCs transcriptome, respectively, with the top DEGs displayed on the left and functional enrichment on the right. C, D Expression levels of candidate genes for prenatal diagnosis in human NTDs transcriptome of brain and spinal cord, respectively. [file 12967_2024_5051_MOESM4_ESM.jpg]

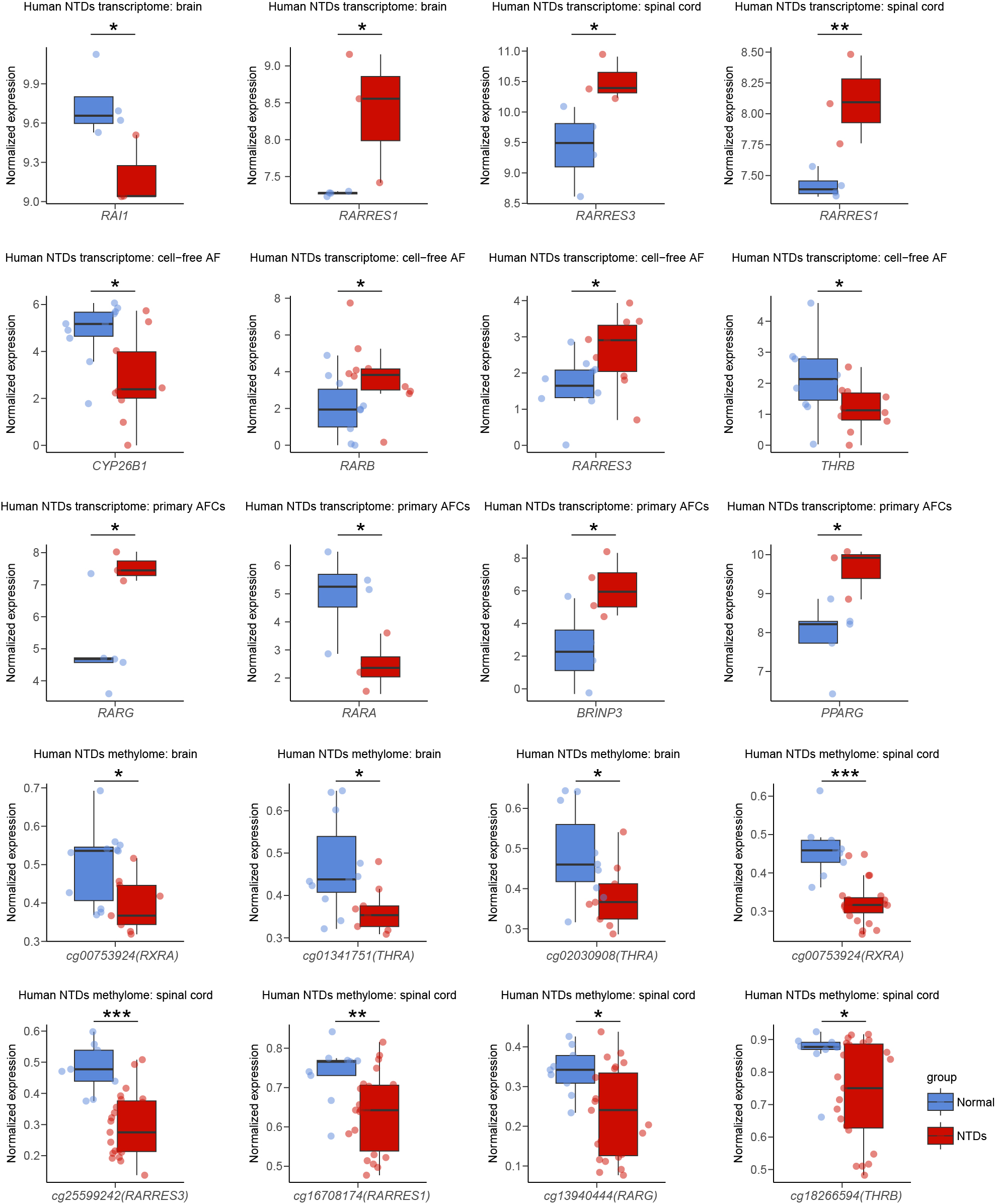

Supplement: Supplementary file 5 — Additional file 5: Figure S5. Dysregulated expression patterns of RA signaling pathway related genes in human NTDs transcriptome and methylome. *P < 0.05; **P < 0.01; ***P < 0.001 [file 12967_2024_5051_MOESM5_ESM.jpg]

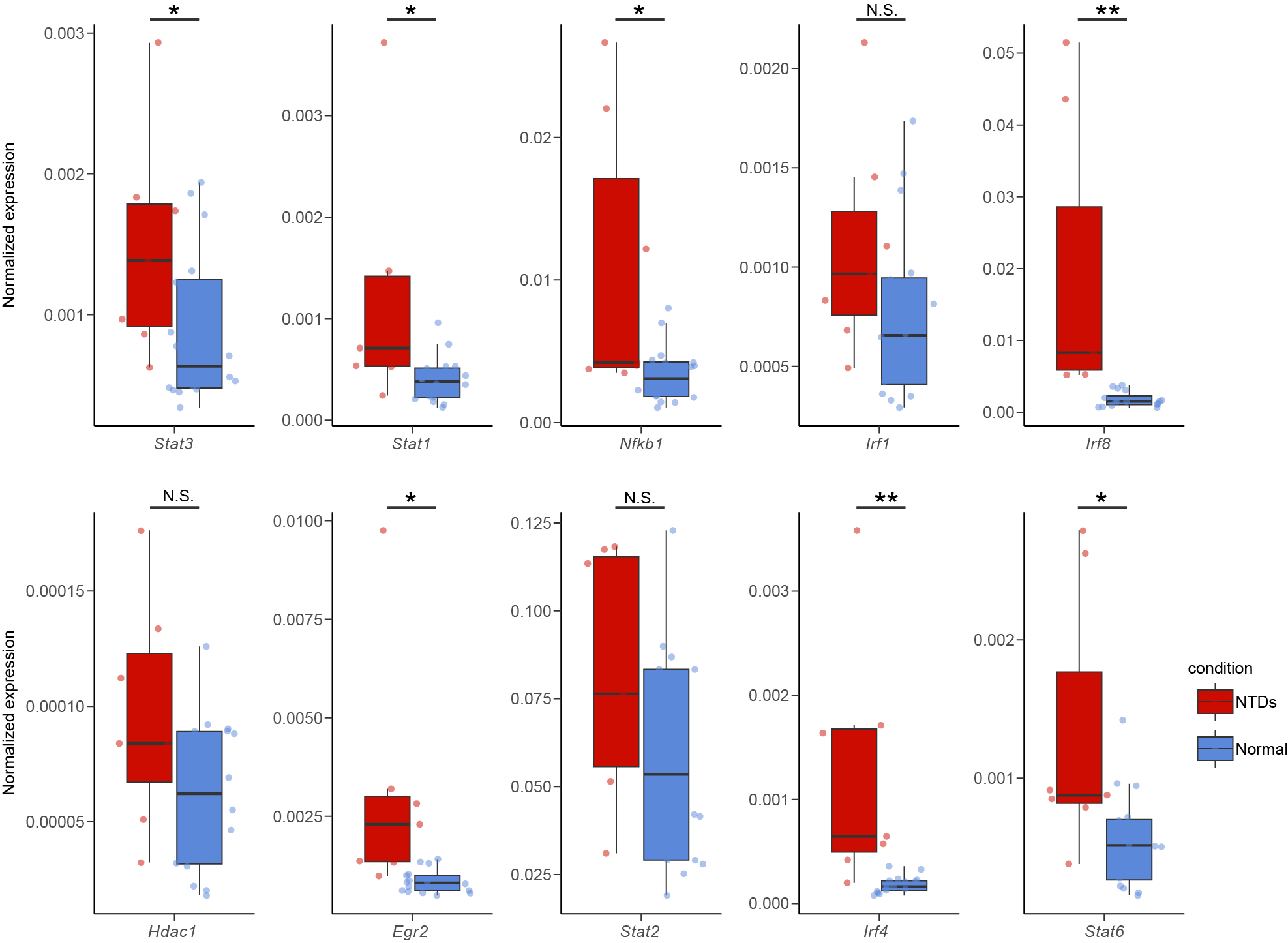

Supplement: Supplementary file 6 — Additional file 6: Figure S6. Expression patterns of pro-inflammatory TFs in RA-induced mouse NTDs amniotic fluid were detected by qPCR assay. *P < 0.05; **P < 0.01, non-significant, N.S. [file 12967_2024_5051_MOESM6_ESM.jpg]

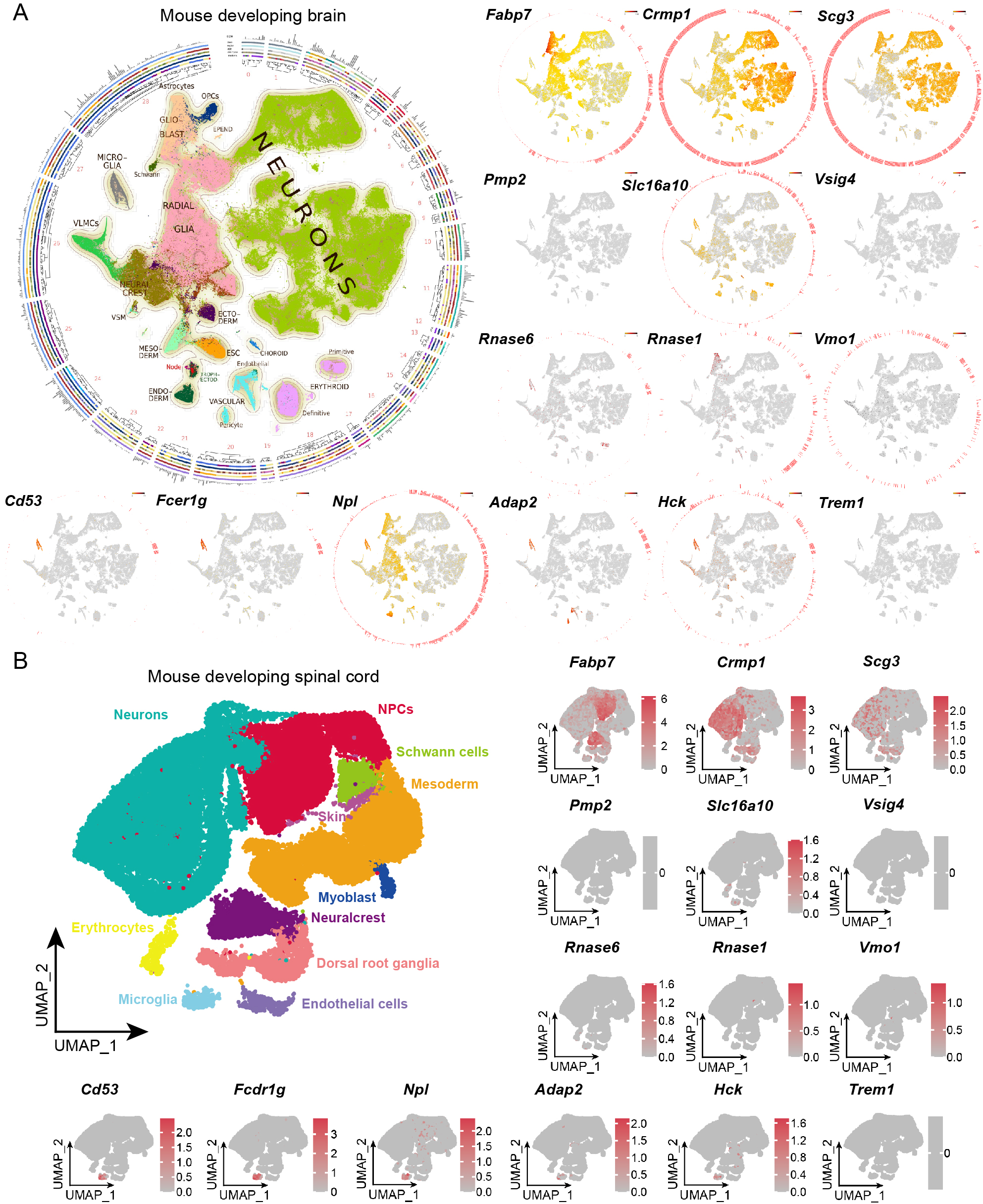

Supplement: Supplementary file 7 — Additional file 7: Figure S7. Expression patterns of candidate genes for prenatal diagnosis in developing mouse brain and spinal cord at single-cell resolution. A Expression patterns of candidate genes for prenatal diagnosis in the developing mouse brain. VLMCs: vascular leptomeningeal cells; VSM: vascular smooth muscle. OPCs: oligodendrocyte progenitors; EPEND: ependymal. B Expression patterns of candidate genes for prenatal diagnosis in the developing mouse spinal cord. NPCs: neural progenitor cells. [file 12967_2024_5051_MOESM7_ESM.jpg]

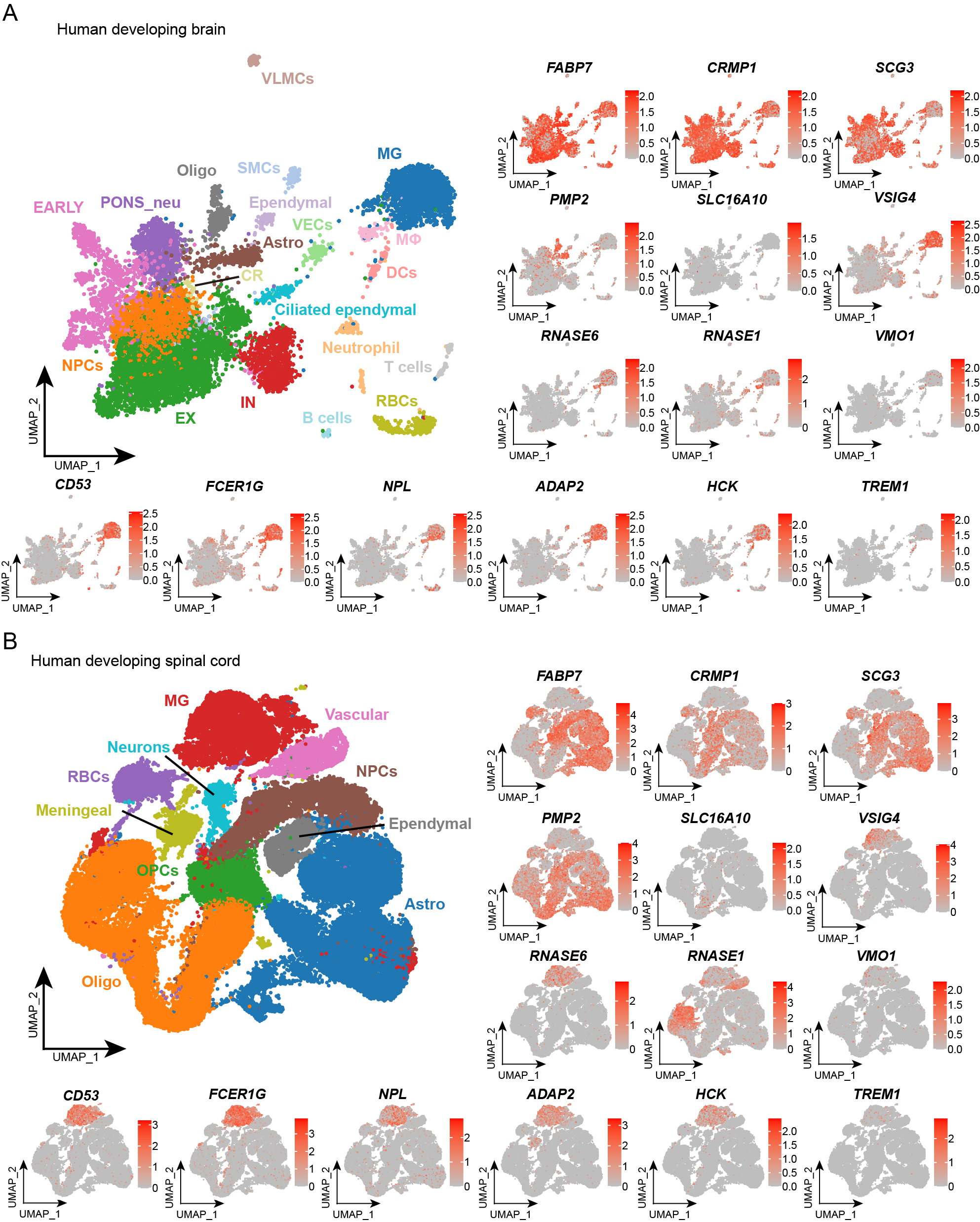

Supplement: Supplementary file 8 — Additional file 8: Figure S8. Expression patterns of candidate genes for prenatal diagnosis in developing human brain and spinal cord at single-cell resolution. A Expression patterns of candidate genes for prenatal diagnosis in the developing human brain. EX: excitatory neuron; CR: Cajal-Retzius cells; IN: inhibitory neuron; Pons-neu: projection neuron in pons; Oligo: oligodendrocytes; Astro: astrocytes; MG: microglia; MΦ: macrophage; SMCs: smooth muscle cells; VECs: vascular endothelial cells; VLMCs: vascular leptomeningeal cells; DCs: Dendritic cells; RBCs: red blood cells. B Expression patterns of candidate genes for prenatal diagnosis in the developing human spinal cord. OPCs: oligodendrocyte progenitors. [file 12967_2024_5051_MOESM8_ESM.jpg]

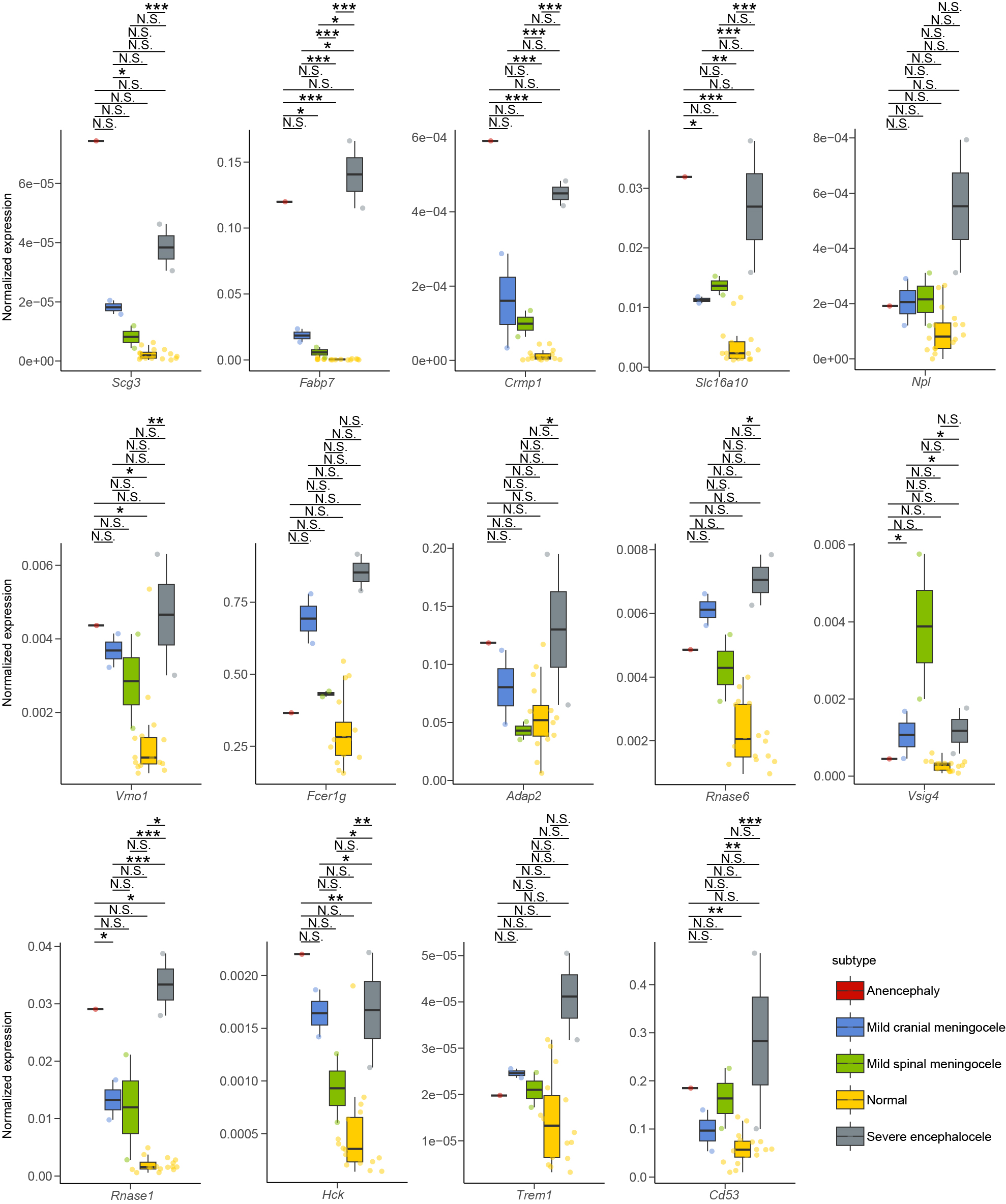

Supplement: Supplementary file 9 — Additional file 9: Figure S9. Expression patterns of candidate prenatal diagnostic markers in four subtypes of the RA-induced mouse NTDs model were examined by qPCR assay. *P < 0.05; **P < 0.01; ***P < 0.001, non-significant, N.S. [file 12967_2024_5051_MOESM9_ESM.jpg]
