# Supplementary material for: Characterizing neuroinflammation and identifying prenatal diagnostic markers for neural tube defects through integrated multi-omics analysis
Source: J Transl Med. 2024 Mar 9;22:257. doi: 10.1186/s12967-024-05051-8 (PMC10924416; doi:10.1186/s12967-024-05051-8)
Supplement: Supplementary file 10 — Additional file 10: Table S1. Primers used for qPCR assay. [file 12967_2024_5051_MOESM10_ESM.docx]

**Additional file 10: Table S1.** Primers used for qPCR assay.

| Gene | Primer sequences |
| --- | --- |
| *Scg3* | Forward: TTGGATACTGGTGTTGGTGCT |
|  | Reverse: TTATGCAGGGATTTGTCTTGGC |
| *Slc16a10* | Forward: GCAGAGAAAGTGGAGGTGGA |
|  | Reverse: TTCTGGATGCCAAACACCGA |
| *Vsig4* | Forward: AATGGCTGGTAAGACACGGC |
|  | Reverse: GTGCAGGGTTGTAGGTGCTT |
| *Rnase1* | Forward: CCCCACCTACTGCAACCAAA |
|  | Reverse: GACATCTGCCAAGGGCTCAT |
| *Npl* | Forward: TTGCTGTCATTGCGCCTTTC |
|  | Reverse: AGCAAAGCAAACTGTCGCTG |
| *Adap2* | Forward: GGCCTCTTACAAGCTGGGG |
|  | Reverse: GTCGTCCCAGAAGTCCAGTC |
| *Vmo1* | Forward: GTGGTACCTGGGGTGATTGG |
|  | Reverse: CGGAGGCAGAAAGCCACTAA |
| *Hck* | Forward: GTTCGAGACTTTGACCCCCA |
|  | Reverse: GGCCATCCACACTTCTCCAA |
| *Trem1* | Forward: CTGCTGTGCGTGTTCTTTGTC |
|  | Reverse: CTGTCAAAGTCTGGCCCTCC |
| *Fabp7* | Forward: GGTTCGGTTGGATGGAGACA |
|  | Reverse: AGTCACGACCATCTTGCCAT |
| *Crmp1* | Forward: CGCCTACGAGAACAAGACCA |
|  | Reverse: TGATGCGTCCACCTCTGATG |
| *Cd53* | Forward: GAGCAGCCTGAAATTGCTGA |
|  | Reverse: CATGCAACCCAAGAAGGCAA |
| *Rnase6* | Forward: CTAAGGGTCTCTCCAGGGCA |
|  | Reverse: TCTTCCGACCGTTCTTGCAG |
| *Fcer1g* | Forward: AGATCCAGGTCCGAAAGGCA |
|  | Reverse: GGGGTGGTTTCTCATGCTTCA |
| *Gapdh* | Forward: ACGGCAAATTCAACGGCACAGT |
|  | Reverse: GGTCTCGCTCCTGGAAGATGGT |
| *Stat1* | Forward: GGAACAGAAAAACGCTGGGAA  Reverse: CTGAAAGGGAACAAAGACCTCC |
| *Stat2* | Forward: CACCTTCCTACTGCGCTTCA  Reverse: GGGGATGTTCTCTTCGGCAA |
| *Stat3* | Forward: AACGACCTGCAGCAATACCA  Reverse: TCCATGTCAAACGTGAGCGA |
| *Stat6* | Forward: GAGCTACTGGTCAGATCGGC  Reverse: GGATGACGTGTGCAATGGTG |
| *Nfkb1* | Forward: AGCAACCAAAACAGAGGGGA  Reverse: TTGCAAATTTTGACCTGTGGGT |
| *Egr2* | Forward: AAGGCCGTAGACAAAATCCCA  Reverse: GCCACTCCGTTCATCTGGTC |
| *Irf1* | Forward: GCAACAGACGAGGATGAGGA  Reverse: CCTATCCCAATGTCCCCTCG |
| *Irf4* | Forward: AGGTGACTCTGTGCTTTGGT  Reverse: GGAAGAATGACGGAGGGAGC |
| *Irf8* | Forward: TTCAAGGCCTGGGCAGTTTT  Reverse: ACAGCGTAACCTCGTCTTCC |
| *Hdac1* | Forward: CTGTGAACTACCCACTGCGA  Reverse: TTGGCGTGTCCTTTGATGGT |
